# Complete mitochondrial genome of Black Soft-shell Turtle (*Nilssonia nigricans*) and comparative analysis with other Trionychidae

**DOI:** 10.1038/s41598-018-35822-5

**Published:** 2018-11-26

**Authors:** Shantanu Kundu, Vikas Kumar, Kaomud Tyagi, Rajasree Chakraborty, Devkant Singha, Iftikar Rahaman, Avas Pakrashi, Kailash Chandra

**Affiliations:** Centre for DNA Taxonomy, Molecular Systematics Division, Zoological Survey of India, M-Block, New Alipore, Kolkata, 700 053 West Bengal India

## Abstract

The characterization of mitochondrial genome has been evidenced as an efficient field of study for phylogenetic and evolutionary analysis in vertebrates including turtles. The aim of this study was to distinguish the structure and variability of the Trionychidae species mitogenomes through comparative analysis. The complete mitogenome (16796 bp) of an endangered freshwater turtle, *Nilssonia nigricans* was sequenced and annotated. The mitogenome encoded for 37 genes and a major non-coding control region (CR). The mitogenome was A + T biased (62.16%) and included six overlapping and 19 intergenic spacer regions. The Relative synonymous codon usage (RSCU) value was consistent among all the Trionychidae species; with the exception of significant reduction of Serine (TCG) frequency in *N. nigricans*, *N. formosa*, and *R. swinhoei*. In *N. nigricans*, most of the transfer RNAs (tRNAs) were folded into classic clover-leaf secondary structures with Watson-Crick base pairing except for *trnS1* (GCT). The comparative analysis revealed that most of the tRNAs were structurally different, except for *trnE* (TTC), *trnQ* (TTG), and *trnM* (CAT). The structural features of tRNAs resulted ≥ 10 mismatched or wobble base pairings in 12 tRNAs, which reflects the nucleotide composition in both H- and L-strands. The mitogenome of *N. nigricans* also revealed two unique tandem repeats (ATTAT)_8,_ and (TATTA)_20_ in the CR. Further, the conserved motif 5′-GACATA-3′ and stable stem-loop structure was detected in the CRs of all Trionychidae species, which play an significant role in regulating transcription and replication in the mitochondrial genome. Further, the comparative analysis of Ka/Ks indicated negative selection in most of the protein coding genes (PCGs). The constructed Maximum Likelihood (ML) phylogeny using all PCGs showed clustering of *N. nigricans* with *N. formosa*. The resulting phylogeny illustrated the similar topology as described previously and consistent with the taxonomic classification. However, more sampling from different taxonomic groups of Testudines and studies on their mitogenomics are desirable for better understanding of the phylogenetic and evolutionary relationships.

## Introduction

The genus *Nilssonia* (Testudines: Trionychidae) was originally described from Myanmar based on *N. formosa* as its type^1^. Further, four species of *Aspideretes* were synonomized under *Nilssonia*^2–4^. At present, the genus *Nilssonia* is known by the five extant species (*N. formosa*, *N. gangetica*, *N. hurum*, *N. leithii*, and *N. nigricans*), which are distributed in India and adjacent countries, from Pakistan, Nepal, Bangladesh, and up to Myanmar. Among them, *N. leithii* is endemic to peninsular India, while *N. formosa* is restricted to Myanmar^5,6^. Nevertheless, the identification of *Nilssonia* congeners has been elusive due to similar conspicuous morphological characters in their different life stages^7,8^. Since the original description, about 150–300 individuals of *N. nigricans* were thought to be living in the shrine pond of Chittagong, Bangladesh that is regarded as type locality^9–11^. Further, due to the countless anecdotal reports on its identification and distribution, *N. nigricans* was categorized as ‘Critically Endangered’ (1996–2000) and later on shifted to ‘Extinct in the wild’ (2002–2018) in the International Union for Conservation of Nature (IUCN, Verson-2017-3) Red data list^12^. Further, in 2011 the IUCN/SSC Tortoise and Freshwater Turtle Specialist Group (TFTSG) drafted the list and categorized the species as Critically Endangered^8^. *N. nigricans* is also included in the Convention on International Trade in Endangered Species of Wild Fauna and Flora (CITES) under ‘Appendix I’ category and recommended to be listed in Indian Wildlife (Protection) Act, 1972. Simultaneously, the species is classified as ‘Endangered’ in the Bangladesh Red List of Reptiles and Amphibians^13^. Various researchers have generated and estimated molecular data to clarify the identification and phylogenetic relationships of *Nilssonia* species^2,6,14–16^. Praschag & Gemel (2002) reported the existence of *N. nigricans* from the state of Assam in northeast India through skull osteology^17^. Further, Praschag *et al*. (2007) and Liebing *et al*. (2012) using partial mitochondrial DNA sequences substantiated the sporadic distribution of *N. nigricans* in Jia Bhoroli river and many temple’s ponds of Assam in northeast India^6,15^. In recent past, Kundu *et al*. 2016 confirmed the wider range distribution of *N. nigricans* in several wild as well as captive localities of Assam and Tripura in northeast India^16^.

The complete mitogenomes have been used to understand the evolutionary relationships of Testudines with other amniotes (reptiles, birds, and mammals)^18,19^. The genome synteny analysis of turtle mitogenome also indicated archosaurian and diapsid affinity with the sister group of the archosaurs (Birds and Crocodilians)^20,21^. Furthermore, gene arrangements in mitogenomes are useful to describe the unusual genomic features and settle the phylogenetic hypotheses for many turtle species^22–25^. So far, the genetic features of the *N. nigricans* mitogenome has remained unknown. Therefore, we sequenced and characterized the complete mitogenome for this species, and compared it with that of other softshell turtles (family Trionychidae) to confirm the unique genetic features and evolutionary relationship. The generated mitogenome will be a valuable resource for further studies on genetic diversity and population structure of *N. nigricans* and also provide new insights for better conservation strategies.

## Materials and Methods

### Sample collection and mitochondrial DNA extraction

The survey was conducted with the prior permission of the wildlife authority of Arunachal Pradesh Biodiversity Board (Letter No. SFRI/APBB/09-2011-1221-1228 dated 22.07.2016); and all experiments were performed in accordance to the relevant guidelines and regulations. The *N. nigricans* specimen was collected from the tributaries of Brahmaputra River in Arunachal Pradesh state (Latitude 27°45′N and Longitude 95°48′E) in northeast India (Fig. S1). The blood sample was collected aseptically by micro-syringe from the hind limb and stored in EDTA vial at 4 °C. After collecting the blood sample, the specimen was released back in the same eco-system with sufficient care. To remove nuclei and cell debris, a drop of blood sample was centrifuged at 700X g for 5 min at 4 °C in 1 ml buffer (0.32 M Sucrose, 1 mM EDTA, 10 mM TrisHCl). The supernatant was collected in 1.5 ml centrifuge tubes and centrifuged at 12000 × g for 10 min at 4 °C to pellet intact mitochondria. The mitochondrial pellet was suspended in 200 µl of lysis buffer (50 mM TrisHCl, 25 mM of EDTA, 150 mM NaCl), with the addition of 10 µl of proteinase K (20 mg/ml) followed by incubation at 56 °C for 1 hour. Finally, the mitochondrial DNA was purified by Qiagen DNeasy Blood & Tissue Kit (QIAGEN Inc.,Germantown MD). The extracted DNA quality was assessed by electrophoresis in a 1% agarose gel stained with ethidium bromide and final concentration was quantified by NANODROP 2000 spectrophotometer (Thermo Scientific, USA).The mitochondrial DNA was deposited in Centre for DNA Taxonomy, Zoological Survey of India, Kolkata under voucher IDs ‘ZSI_NFGR-TT8’.

### Genome sequencing, assembly and annotation

The complete mitochondrial genome sequencing, *de novo* assembly and annotation were carried out at the Genotypic Technology Pvt. Ltd. Bangalore, India (http://www.genotypic.co.in/). The whole genome library was sequenced using the Illumina NextSeq 500 (150 × 2 chemistry) (Illumina, Inc, USA) which yielded total 4677068 raw reads. The raw reads were processed using cutadapt tool (http://code.google.com/p/cutadapt/) for adapters and low quality bases trimming with cutoff of Phread quality score (Q score) of 20. These high quality reads were down sampled to 2 million reads using Seqtk program (https://github.com/lh3/seqtk) and the reads were *de novo* assembled using SPAdes-3.7.1. using default parameters^26^, considering *Pelochelys cantorii* mitochondrial genome (JN016746) as reference contig. The sequence annotation was also checked in MITOS online server (http://mitos.bioinf.uni-leipzig.de). The nucleotide sequences of the protein coding genes (PCGs) were initially translated into the putative amino acid sequences on the basis of the vertebrate mitochondrial DNA genetic code. The exact initiation and termination codons were identified in ClustalX using other reference sequences of Testudines^27^.

### Genome sequence and Phylogenetic analysis

The direction of locus, size, start and stop codon of PCGs, overlapping regions and intergenic spacers between genes, anticodon of transfer RNA (tRNA) genes were checked in MITOS online server (http://mitos.bioinf.uni-leipzig.de) and Open Reading Frame Finder (https://www.ncbi.nlm.nih.gov/orffinder/). The PCGs located in light strand (L strand) were made reverse complementary before incorporating them in the analysis. However, the nucleotide composition and ATGC- skew of tRNA genes, situated in both heavy strand (H strand) and light strand (L strand) were analyzed as its original orientation. The CGView Server (http://stothard.afns.ualberta.ca/cgview_server/) with default parameters was used to map the circular representation of the generated completed mitochondrial genome^28^. Based on the homology search against the Refseq mitochondrial database (https://www.ncbi.nlm.nih.gov/refseq/), 12 mitogenomes of Trionychidae species were acquired from GenBank and incorporated in the dataset for comparative analysis. The base composition of nucleotide sequences, and composition skewness were calculated manually in Microsoft Excel as described previously: AT skew = [A − T]/[A + T], GC skew = [G − C]/[G + C]^29^. The Relative Synonymous Codon Usage (RSCU) values of each PCGs were calculated by assuring their codon frame using MEGA6.0^30^. The tRNA genes were verified in MITOS online server (http://mitos.bioinf.uni-leipzig.de), tRNAscan-SE Search Server 2.0 (http://lowelab.ucsc.edu/tRNAscan-SE/) and ARWEN 1.2 with the default settings with the appropriate anticodon capable of folding into the typical cloverleaf secondary structure^31,32^. The base composition of all stems (DHU, acceptor, TψC, and anticodon) were checked manually to distinguish the Watson-Crick, wobble, and mismatched base pairing. The large and small subunit of ribosomal RNA (*rrnL* and *rrnS*) were annotated by the MITOS online server (http://mitos.bioinf.uni-leipzig.de). In order to determine the unique base compositions in the control regions (CRs) of 13 Trionychidae species including *N. nigricans*, tandem repeats were predicted by the online Tandem Repeats Finder web tool (https://tandem.bu.edu/trf/trf.html)^33^. Subsequently, the CRs of 13 Trionychidae species were aligned using ClustalX software and checked manually in order to define the conserved sequence blocks and conserved sequence motif^27^. To recognize the location for replication of the L-strand, putative secondary structure of the CRs were anticipated by the online Mfold web server (http://unafold.rna.albany.edu)^34^. The software package DnaSPv5.0 was used to calculate the synonymous substitutions per synonymous sites (Ks) and non-synonymous substitutions per non-synonymous sites (Ka)^35^. Further, the complete mitogenomes of five turtle species under five different families (suborder: Cryptodira) were also incorporated to the dataset to illustrate the phylogenetic relationships. The complete mitogenome of *Chelus fimbriata*, family Chelidae (suborder: Pleurodira) was incorporated as an out-group in the phylogenetic analysis. The PCGs were individually aligned with the TranslatorX online platform using the MAFFT algorithm with the GBlocks parameters and default settings^36^. The dataset of all PCGs was concatenated using SequenceMatrix v1.7.845^37^. To construct the Maximum Likelihood (ML) tree, the best model ‘GTR + G + I’ was selected with the lowest Bayesian information criterion (BIC) scores (158167.27) by using Maximum Likelihood statistical method in MEGA6.0 program^30^.

## Results and Discussion

### Genome size and organization

**Figure 1:**
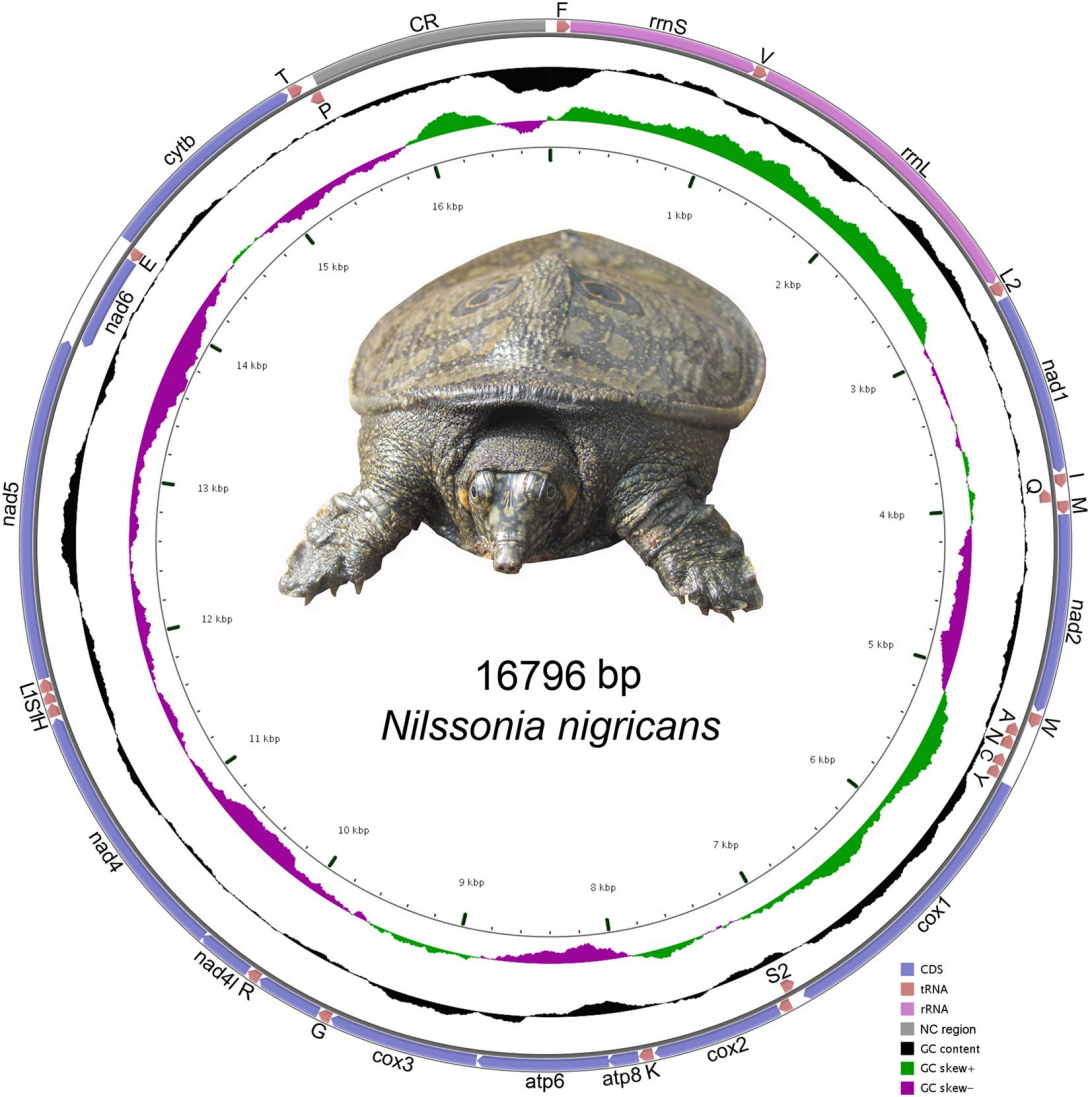
The mitochondrial genome of *N. nigricans*. Direction of gene transcription is indicated by arrows. PCGs are shown as blue arrows, rRNA genes as orchid arrows, tRNA genes as coral arrows and 1290 bp non coding region as grey rectangle. tRNAs are encoded according to their single-letter abbreviations. The GC content is plotted using a black sliding window, GC-skew is plotted using green and violet color sliding window as the deviation from the average in the complete mitogenome. The figure was drawn using CGView online server (http://stothard.afns.ualberta.ca/cgview_server/) with default parameters. The Species photographs was taken by the first authors (S.K.) by using Nikon D3100 and edited manually in Adobe Photoshop CS 8.0.

In this study, the closed-circular complete mitogenome (16796 bp) of the black soft-shell turtle, *N. nigricans* was determined under the ‘National Faunal Genome Resources (NFGR)’ program at Zoological Survey of India, Kolkata (Fig. 1). The generated mitogenome was submitted to the GenBank database through Sequin submission tool (https://www.ncbi.nlm.nih.gov/projects/Sequin/) and acquired the accession number: MG383833. The comparative analysis showed the length of mitogenomes ranging from 16489 bp in *L. punctata* to 17499 bp in *P. cantorii*. All the 37 genes were annotated in the *de novo* assembled genome; 13 PCGs, 22 tRNAs, two ribosomal RNAs (rRNAs), and a major non-coding CR (Table 1). Among them, 28 genes (12 PCGs, two rRNAs, and 14 tRNAs) were located on the heavy strand (H strand) and others (*nad6* and eight tRNAs) were located on the light strand (L strand). The organization of genes in *N. nigricans* mitogenome resulted in a similar arrangement as is described for typical vertebrate species^38^. The comparative analysis revealed that similar gene arrangements in both L and H strand were present in other Trionychidae species (Table S2). The total length of PCGs, tRNAs, and rRNAs was 11251 bp, 1551 bp, and 2593 bp respectively. The nucleotide composition was biased toward A + T (62.16%) within the complete mitogenome of *N. nigricans*. The A + T composition was 61.26%, 63.82%, 61.47%, and 68.52% in PCGs, tRNAs, rRNAs and CR respectively (Table S1). In other Trionychidae species, the nucleotide composition within the complete mitogenomes is similar to *N. nigricans* and biased towards A + T with a variable frequency ranging from 58.48% (*T. triunguis*) to 62.97% (*N. formosa*). In the complete mitogenome of *N. nigricans*, the AT and GC skew was 0.197 and −0.400 respectively. The AT skew of other Trionychidae species varied from 0.127 (*P. sinensis*) to 0.208 (*T. triunguis*) and GC skew were varied from −0.412 (*T. triunguis*) to −0.363 (*P. steindachneri*). The positive AT skew in most of the genes indicated more Adenine (A)s than Thymine (T)s in the complete mitogenomes of Trionychidae species (Table S1).

**Table 1:** List of annotated mitochondrial genes of *Nilssonia nigricans*.

Locus Name	Direction	Location	Size (bp)	Anti codon	Start codon	Stop codon	Intergenic Nucleotides
*trnF*	**+**	59–128	70	GAA	**—**	**—**	0
*rrnS*	**+**	129–1110	982	**—**	**—**	**—**	**−**2
*trnV*	**+**	1109–1178	70	TAC	**—**	**—**	0
*rrnL*	**+**	1179–2789	1611	**—**	**—**	**—**	0
*trnL2*	**+**	2790–2865	76	TAA	**—**	**—**	13
*nad1*	**+**	2879–3823	945	**—**	ATA	A	8
*trnI*	**+**	3832–3901	70	GAT	**—**	**—**	**−**1
*trnQ*	**−**	3901–3971	71	TTG	**—**	**—**	**−**1
*trnM*	**+**	3971–4039	69	CAT	**—**	**—**	0
*nad2*	**+**	4040–5068	1029	**—**	ATG	A	13
*trnW*	**+**	5082–5156	75	TCA	**—**	**—**	4
*trnA*	**−**	5161–5229	69	TGC	**—**	**—**	1
*trnN*	**−**	5231–5304	74	GTT	**—**	**—**	33
*trnC*	**−**	5338–5403	66	GCA	**—**	**—**	0
*trnY*	**−**	5404–5471	68	GTA	**—**	**—**	4
*cox1*	**+**	5476–7008	1533	**—**	ATT	A	4
*trnS2*	**−**	7013–7083	71	TGA	**—**	**—**	0
*trnD*	**+**	7084–7152	69	GTC	**—**	**—**	0
*cox2*	**+**	7153–7833	681	**—**	ATG	T	7
*trnK*	**+**	7841–7916	76	TTT	**—**	**—**	1
*atp8*	**+**	7918–8076	159	**—**	ATG	A	**−**4
*atp6*	**+**	8073–8753	681	**—**	ATG	(TAA)	2
*cox3*	**+**	8756–9538	783	**—**	ATG	T	1
*trnG*	**+**	9540–9609	70	TCC	**—**	**—**	0
*nad3*	**+**	9610–9958	348	**—**	ATG	AGA	1
*trnR*	**+**	9960–10030	71	TCG	**—**	**—**	1
*nad4l*	**+**	10032–10325	294	**—**	ATG	(TAA)	**−**4
*nad4*	**+**	10322–11692	1371	**—**	ATG	(C)	10
*trnH*	**+**	11703–11772	70	GTG	**—**	**—**	0
*trnS1*	**+**	11773–11834	62	GCT	**—**	**—**	**−**1
*trnL1*	**+**	11834–11905	72	TAG	**—**	**—**	0
*nad5*	**+**	11906–13681	1776	**—**	ATG	(TAA)	1
*nad6*	**−**	13683–14204	522	**—**	ATG	(T)	0
*trnE*	**−**	14205**−**14272	68	TTC	**—**	**—**	9
*cytb*	**+**	14282–15409	1128	**—**	ATC	A	9
*trnT*	**+**	15419–15492	74	TGT	**—**	**—**	2
*trnP*	−	15495–15564	70	TGG	**—**	**—**	0
A + T-rich Region		15565–16796 1–58	1290	**—**	**—**	**—**	**—**

### Overlapping and intergenic spacer regions

Six overlapping sequences with a total length of 13 bp were identified in the complete mitogenome of *N. nigricans* (Table 1). These sequences varied in length (1 bp to 4 bp) with the longest overlapping region present between NADH dehydrogenase subunit 4 L (*nad4l*) and NADH dehydrogenase subunit 4 (*nad4*) as well as in between ATP synthase F0 subunit 8 (*atp8*) and ATP synthase F0 subunit 6 (*atp6*). Further, the intergenic spacers within this mitogenome, spread over 19 regions and ranged in size from 1 bp to 33 bp with a total length of 124 bp. The longest spacer (33 bp) occurred between *trnN* (GTT) and *trnC* (GCA). The comparative analysis showed the number of overlapping sequences varied from five (*N. formosa*) to 14 (*P. sinensis*) with a length variation of 7 bp to 143 bp in other Trionychidae species. The longest intergenic spacer (33 bp) is present between *trnN* (GTT) and *trnC* (GCA) of *L. scutata* and *L. punctata* (Table S3).

### Protein-coding genes (PCGs) features

The PCGs of *N. nigricans* were 11251 bp long and shares 66.9% of the complete mitogenome. The contrast of nucleotide composition, AT skew and GC skew of all Trionychidae species is shown in Table S1. The AT skew was 0.136 and the GC skew was −0.414 in *N. nigricans*. The comparative analysis showed that, the AT skew of other Trionychidae species varied from 0.069 (*P. sinensis*) to 0.138 (*T. triunguis*) and GC skew from −0.450 (*L. scutata*) to −0.370 (*P. sinensis*) (Table S1). Further, most of the the PCGs of *N. nigricans* were started by ATG initiation codon, except for NADH dehydrogenase subunit 1 (*nad1*) by ATA, Cytochrome oxidase subunit I (*cox1*) by ATT, and Cytochrome b (*cytb*) by ATC. Out of 13 PCGs, three PCGs (*atp6*, *nad4l*, *nad5*) were used a typical (TAA) termination codon; whereas the remaining three PCGs (*cox2*, *cox3*, *nad6*) terminated by a single base (T), five PCGs (*nad1*, *nad2*, *cox1*, *atp8*, *cytb*) terminated by a single base (A), *nad3* terminated by AGA, and *nad4* terminated by a single base (C) (Table 1). The comparative analysis of the studied Trionychidae species revelaed that most of the PCGs were started by ATN initiation codons. Majority of the PCGs were started by ATG, except ATT for *cox1* (*N. formosa*), ATC for *cytb* (*N. formosa*), GTG for *cox1* (*D. subplana*, *P. steindachneri*, *P. sinensis*, *R. swinhoei*, *A. spinifera*, *A. ferox*, *T. triunguis*, *C. indica*, *P. cantorii*, *L. scutata*, *L. punctata*) and *nad5* (*L. scutata*), ATA for *nad1* (*P. steindachneri*) and *nad6* (*P. steindachneri*, *A. spinifera*, *A. ferox*, *C. indica*), ATT for *cytb* (*P. sinensis*). The termination codons were TAA for most of the Trionychidae species; except for single base (T) for *nad1* (*N. formosa*), *cox3* and *nad3* (*N. formosa*, *D. subplana*, *P*. steindachneri, *A. spinifera*, *R. swinhoei*, *A. ferox*, *C. indica*, *P. cantorii*, *L. scutata*, *L. punctata*), *nad2* and *nad4* (*P. steindachneri*, *A. spinifera*, *A. ferox*, *T. triunguis*, *C. indica*, *P. cantorii, L. scutata*, *L. punctata*), *nad4* (*D. subplana*, *R. swinhoei*), *nad4l* (*D. subplana*), *cytb* (*R. swinhoei*, *A. ferox*), *cox3* (*T. triunguis*). The termination codon (TA) was observed in *nad1* (*P. steindachneri*, *R. swinhoei, A. spinifera*, *A. ferox*), *nad2* and *nad4l* (*R. swinhoei*). Further, the termination codon AGA was observed in *nad6* (*D. subplana*, *C. indica*, *L. punctata*, *P. steindachneri*, *R. swinhoei*, *A. spinifera*, *A. ferox*, *P. cantorii*, *L. scutata*, *T. triunguis*), *cox1* (*P. steindachneri*, *R. swinhoei*, *A. spinifera*, *A. ferox*, *P. cantorii*, *L. scutata*, *P. sinensis*), *nad3* (*T. triunguis*). The termination codon AGG was used by *cox1* (*C. indica*), and *nad6* (*P. sinensis*). The termination codon TAG was used by *nad1* (*D. subplana*, *P. sinensis*, *L. punctata*), *nad2* (*D. subplana*, *P. sinensis*), and *nad5* (*L. scutata*) (Table S4). The codons of each amino acids were conserved in all PCGs of the compared Trionychidae species. Moreover, codons with A or T in the third position were overused in comparison to other synonymous codons. For example, the codon for Glutamine (Gln) as CAG was rare as compared to CAA; and codon for Glutamic acid (Glu) as GAG was rare as compared to GAA. The comparative RSCU analysis indicated a significant fall in frequency of the TCG codon in Serine (Ser) in *N. nigricans*, *N. formosa*, and *R. swinhoei* (Table S5). The RSCU analysis of *N*. *nigricans* revealed that, the codons encoding for Asparagine (Asn), Histidine (His), Isoleucine (Ile), Leucine (Leu), Methionine (Met), Threonine (Thr), Proline (Pro), and Serine (Ser) were the most frequently present, nevertheless those encoding Arginine (Arg), Aspartic acid (Asp), Cysteine (Cys), and Glutamic acid (Glu) were rare. The RSCU analysis showed the same frequency of codon usage for their respective amino acids in other Trionychidae species.

### Transfer RNA (tRNAs) and ribosomal RNA (rRNAs)

The representative forecast structures of 22 tRNAs, total of 1551 bp were identified in *N. nigricans* mitogenome, ranging from 62 bp to 76 bp. Among them, 14 were present in H-strand and the remaining eight were present in L-strand (Table 1). The comparative analysis with other Trionychidae species depicted the total length of tRNAs varied from 1540 bp (*D. subplana* and *R. swinhoei*) to 1571 bp (*P. sinensis*). The AT skew in *N. nigricans* was 0.143 and GC skew was −0.215. The comparative results showed the AT skew varied from 0.111 (*P. sinensis*) to 0.159 (*T. triunguis*) and GC skew varied from −0.226 (*P. cantorii and T. triunguis*) to −0.184 (*P. steindachneri*) (Table S1). The comparative results showed that the anticodons for all tRNAs were similar in Trionychidae species (Table S6). It has been suggested that the unique arrangements in the ‘WANCY’ region in vertebrates, have a significant role to fold a secondary structure and replace the function of light strand in mitogenome^39^. Similar tRNAs arrangements were also shared by *N. nigricans* and other Trionychidae species. Both rRNA genes (*rrnS* and *rrnL*) were located in the H-strand in *N. nigricans* and other Trionychidae species. The total length of small and large rRNA genes was 2593 bp with AT skewness 0.307 and GC skewness −0.180. The comparative analysis with other Trionychidae species showed, the length of two rRNAs varied from 2546 bp (*L. punctata*) to 2685 bp (*P. sinensis*). The AT skew varied from 0.268 (*P. sinensis*) to 0.329 (*T. triunguis*) and GC skew varied from −0.216 (*T. triunguis*) to −0.145 (*L. punctata*). The *rrnS* gene is located in between the *trnF* (GAA) and *trnV* (TAC); however, the *rrnL* gene is located in between *trnV* (TAC) and *trnL2* (TAA) in *N. nigricans* as also occurs in other Trionychidae species (Table S1).

### Comparision of tRNAs secondary structure

**Figure 2:**
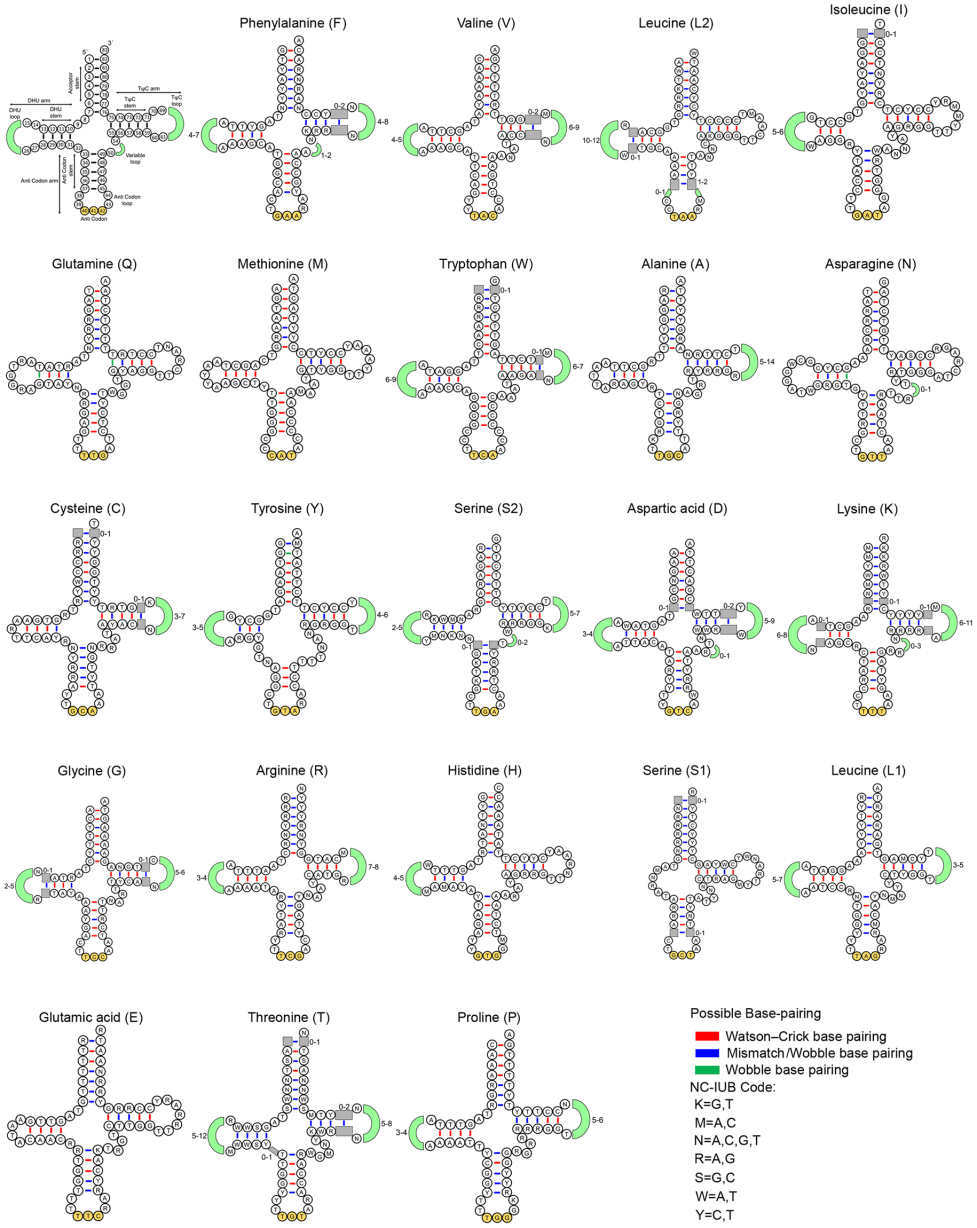
Secondary structures of 22 transfer RNAs (tRNAs) displaying the structural variation in the 13 Trionychidae species. The first structure shows the nucleotide positions and details of stem-loop of tRNAs. The tRNAs are represented by full names and IUPAC-IUB single letter amino acid codes. Stems and loops with length variation are marked by gray squares and light green arcs respectively. Watson-Crick, wobble, and mismatch base pairing are shown by red, green and blue color bars respectively. The secondary structure of tRNAs were predicted by MITOS online server (http://mitos.bioinf.uni-leipzig.de/index.py) and edited manually in Adobe Photoshop CS 8.0.

The wobble base pairing is a key characteristic of RNA structure and often susbtitutes the GC or AT base pairs due to thermodynamic stability. These features play essential functional roles in a wide range of biological processes^40^. RNA-binding proteins adhere to unique G-U sites by recognizing chemical features and differ from Watson-Crick and other mismatched pairs^41^. Thus, the extensive comparisons of tRNAs are crucial for understanding the structural and functional features of the mitogenomes^42^. In *N. nigricans*, most of the tRNAs were folded into classic clover-leaf secondary structures, except for *trnS1* (GCT). Similar characteristics were also present in all other Trionychidae species (Fig. 2). The tRNA secondary structures of *N. nigricans*, the Watson-Crick base pairing were observed in most of the positions. The highest changes of base pairing were observed in *trnQ* (TTG) and *trnA* (TGC), while no changes has been detected in *trnK* (TTT) and *trnL1* (TAG). Further, the wobble base pairings were observed in 10 tRNAs: in the acceptor stem of *trnQ*, *trnA*, *trnN*, *trnC*, *trnY*, *trnS2*, and *trnE*; in the TψC stem of *trnV*, *trnQ*, *trnA*, *trnS2* and *trnP*; in the anticodon stem of *trnA*, *trnS2*, *trnE*, and *trnP*; and in the DHU stem of *trnN* and *trnG*. The comparative analysis of 13 Trionychidae species revealed that most of the tRNAs were structurally different in both stem and loop. However three tRNA genes, *trnE* (TTC), *trnQ* (TTG), and *trnM* (CAT) showed similar secondary structure in all 13 Trionychidae species. The DHU stem and loop was absent in *trnS1* (GCT), while the variation in base composition were observed in the variable loop of *trnN*, *trnF*, *trnS2*, *trnD*, and *trnK*. The comparative analysis revealed prominent wobble base pairing in *trnQ* (DHU and TψC stem), *trnN* (DHU stem), and *trnY* (acceptor stem). The comparative structural features also resulted, ≥10 mismatched or wobble base pairings were observed in 12 tRNAs with highest in *trnT* and lowest in *trnV* (Fig. 2). Thus, the resultant alteration in base composition of tRNAs reflects the nucleotide composition in both H- and L-strands.

### Control region (CR)

**Figure 3:**
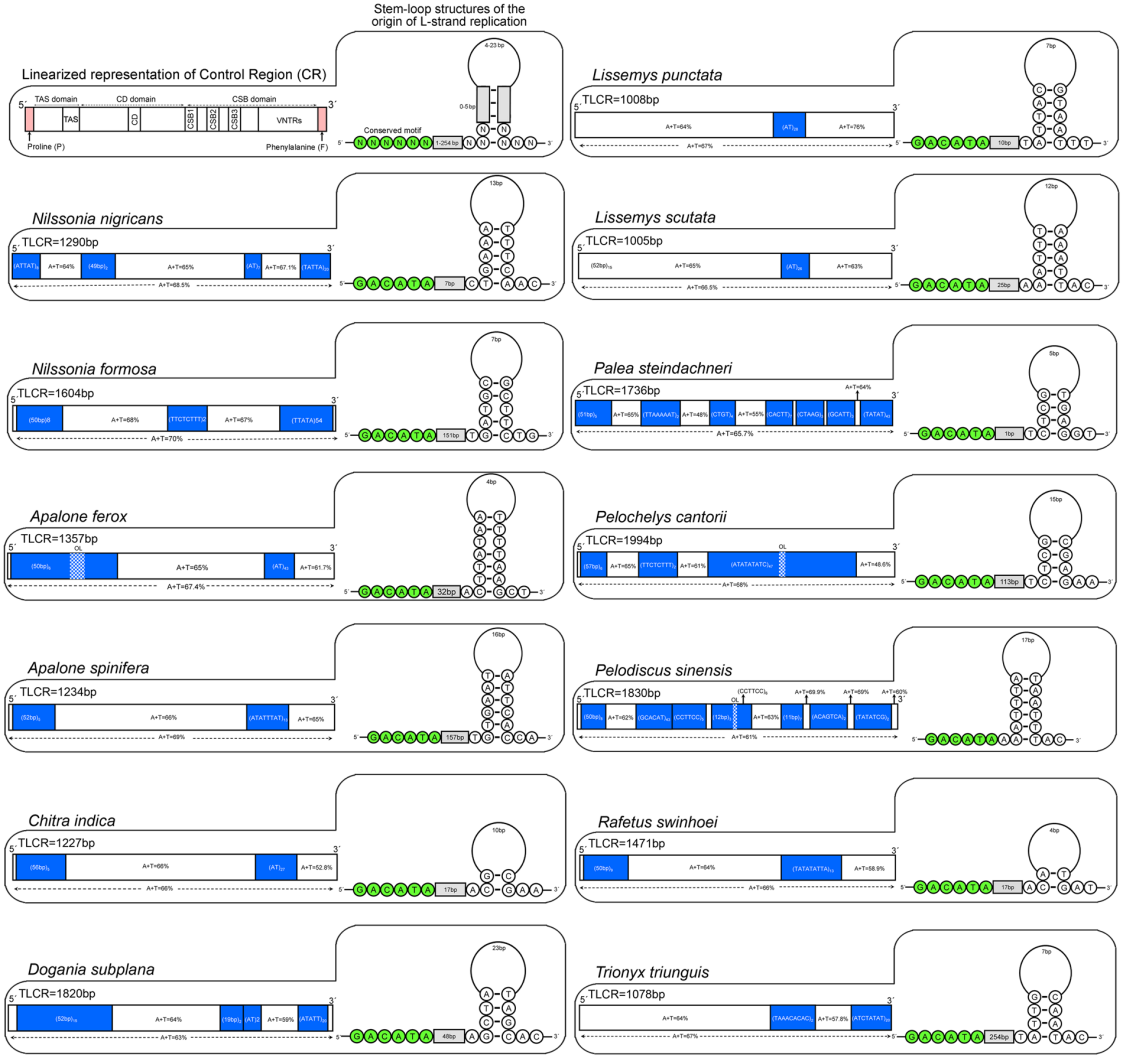
Comparison of length, nucleotide composition in control regions (CRs) and stem-loop structures of 13 Trionychidae species. The first structure shows the linearized representation of CR and stem-loop structures of the origin of L-strand replication. Blue color boxes shows the tandem repeats within the CR. OL = Overlapping regions are marked by blue-white pattern. TLCR = Total length of the CR. Nucleotides marked by green circles represent the conserved sequence motif. The tandem repeats were predicted by the online Tandem Repeats Finder web tool (https://tandem.bu.edu/trf/trf.html) and the putative secondary structures were predicted by the online Mfold web server (http://unafold.rna.albany.edu). The figure was edited manually in Adobe Photoshop CS 8.0.

The mitochondrial control region (CR) is generally divided into three functional domains: the termination associated sequence (TAS) domain, the central conserved domain (CD), and the conserved sequence block (CSB) domain^43^. The CD domain comprises more highly conserved sequences than TAS and CSB domain with variable numbers of tandem repeats (VNTRs) and origin of the H-strand transcription^44^. The pattern of conserved sequences vary among different vertebrate groups: humans^45^, birds^46^, and reptiles including turtles^47–50^. The length of CR was 1290 bp and the A + T content was 68.52% in *N. nigricans* (Table S1). Three conserved blocks (CSB-1, CSB-2, and CSB-3) were depicted in the CSB domains of *N. nigricans* CR, as similar to other turtle species of suborder Cryptodira^51,52^. It is reported that, the CSB-2 and CSB-3 were absent in the CRs of Pleurodira species^44^. These unique features suggest that the regulatative mechanisms of transcription might vary between Cryptodira and Pleurodira suborders, and could provide significant diagnostic characters. Further four VNTRs; (ATTAT)_8_, (49 bp)2, (AT)_7_, and (TATTA)_20_ were observed in *N. nigricans* CR with spacer of 151 bp was observed between 1^st^ and 2^nd^ tandem repeat; 781 bp between 2^nd^ and 3^rd^ tandem repeat, and 87 bp between 3^rd^ and 4^th^ tandem repeat (Fig. 3). The AT skew value was positive (0.07) and GC skew value was negative (−0.374). The length of the CR varied from 1005 bp (*L. scutata*) to 1994 bp (*P. cantorii*) in the other Trionychidae species. The AT and GC skew in other Trionychidae species have significant variation and range from −0.106 (*P. steindachneri*) to 0.067 (*T. triunguis*) and −0.453 (*P. cantorii*) to −0.230 (*P. steindachneri*) respectively. As a result, Adenine (A) composition is higher than Thymine (T) in *N. nigricans* in comparison with other Trionychidae species (*C. indica*, *L. punctata*, *L. scutata*, *P. cantorii*, and *T. triunguis*). However, Thymine (T) composition is higher than Adenine (A) in *A. ferox*, *A. spinifera*, *D. subplana*, *N. formosa*, *P. steindachneri*, *P. sinensis*, and *R. swinhoei* (Table S1). The frequency of tandem repeats is higher at the 3ʹ end of the CR in all Trionychidae species. The numbers VNTRs were varied from two (*A. spinifera*, *C. indica*, *R. swinhoei*, and *T. triunguis*) to eight (*P. sinensis*). A single short tandem repeat (STRs) of AT was found in two Trionychidae species (*L. scutata*, and *L. punctata*). The length of the tandem repeats was variable in other Trionychidae species, ranging from 2 bp (*A. ferox*, *C. indica*, *D. subplana*, *L. scuata*, *L. punctata*) to 57 bp (*P. cantorii*). Exceptionally, overlapping of tandem repeats was found in *A. ferox*, *P. cantorii*, and *P. sinensis*. Comparative analysis revealed a single tandem repeat of (AT)_43_, (ATATTTAT)_13_, (AT)_27_ and (TATATATTA)_13_ in *A. ferox*, *A. spinifera*, *C. indica*, and *R. swinhoei* respectively. Exceptionally, in *T. triunguis*, *L. scutata*, and *L. punctata* tandem repeats were lacking at the 5′ end of CR in comparison with other species. Further, two tandem repeats (TAAACACAC)_2_ and (ATCTATAT)_20_ with intergenic spacer of 71 bp was observed in *T. triunguis* and one tandem repeat of (AT)_26_ and (AT)_28_ was observed in *L. scutata* and *L. punctata* respectively (Fig. 3). Further, the origin of L-strand replication was identified in 13 Trionychidae species including *N. nigricans*. The conserved motif 5′-GACATA-3′ in CSB-1 block and stable stem-loop structure was identified in *N. nigricans* and other Trionychidae species CRs as identical among other vertebrates^46,53^. The stem and loop lengths ranged from 2 to 7 bp and from 4 to 23 nucleotides, respectively (Fig. 3). The structural features, and duplication of CR play an important role in regulating transcription and replication in the mitochondrial genome^54,55^. The present study evaluated the genetic features of CR among the studied Trionychidae species mitogenomes including *N. nigricans* that will be helpful to hypothesize the evolutionary pattern of this group.

### Synonymous and non-synonymous substitutions

Darwinian selection is an important hypothesis for recognizing the evolutionary pattern of positively selected genes and plays an important role behind species divergence^56,57^. The analysis of mitogenome for detecting positive selection of PCGs serve as supportive technique for understanding the influences of natural selection in evolution and amending protein function^58–61^. The comparison of synonymous (Ks) and nonsynonymous (Ka) substitution rates in PCGs, evidenced for darwinian selection and adaptive molecular evolution in vertebrates^62–64^. The ratio of Ka/Ks was generally known as an indicator of selective pressure and evolutionary relations at molecular level among the homogenous or heterogeneous species^65^. It is reported that, for positive selection Ka/Ks > 1, for neutrality Ka/Ks = 1, and for negative selection Ka/Ks < 1^66^. This approach has the benefit to reveal the natural selection acting on PCGs. Thus, to investigate the evolutionary rates between homologous gene pairs, Ka/Ks substitutions were calculated and compared with 10 other Trionychidae species of 10 representative genera. The average Ka/Ks values of 13 PCGs varied from 0.042 ± 0.013 (*cox3*) to 1.129 ± 0.274 (*nad4l*) and resulted in the following order: *cox3* < *cox2* < *atp6* < *nad6* < *nad3* < *atp8* < *cox1* < *nad5* < *nad2* < *nad4* < *cytb* < *nad1* < *nad4l* (Table S7). Most of the PCGs show Ka/Ks values of <1, which indicated a strong negative selection among the studied Trionychidae species, that reflects natural selection works against deleterious mutations with negative selective coefficients as highlighted general patterns in other vertebrates^62,65^. The average Ka/Ks variation was >1 in *cytb*, *nad1* and *nad4l* with values of 1.051, 1.120 and 1.129 respectively, which indicated the least selective pressure or positive selection in respective PCGs. Among all PCGs, average of Ka/Ks was lowest for *cox3* (0.042) indicating negative selection. Thus, comparative analysis of Ka/Ks in Trionychidae species mitogenomes will facilitate understanding the natural selection that influences the evolution of species, interaction between mutations and selective pressures that are responsible for protein evolution.

### Phylogenetic analysis

**Figure 4:**
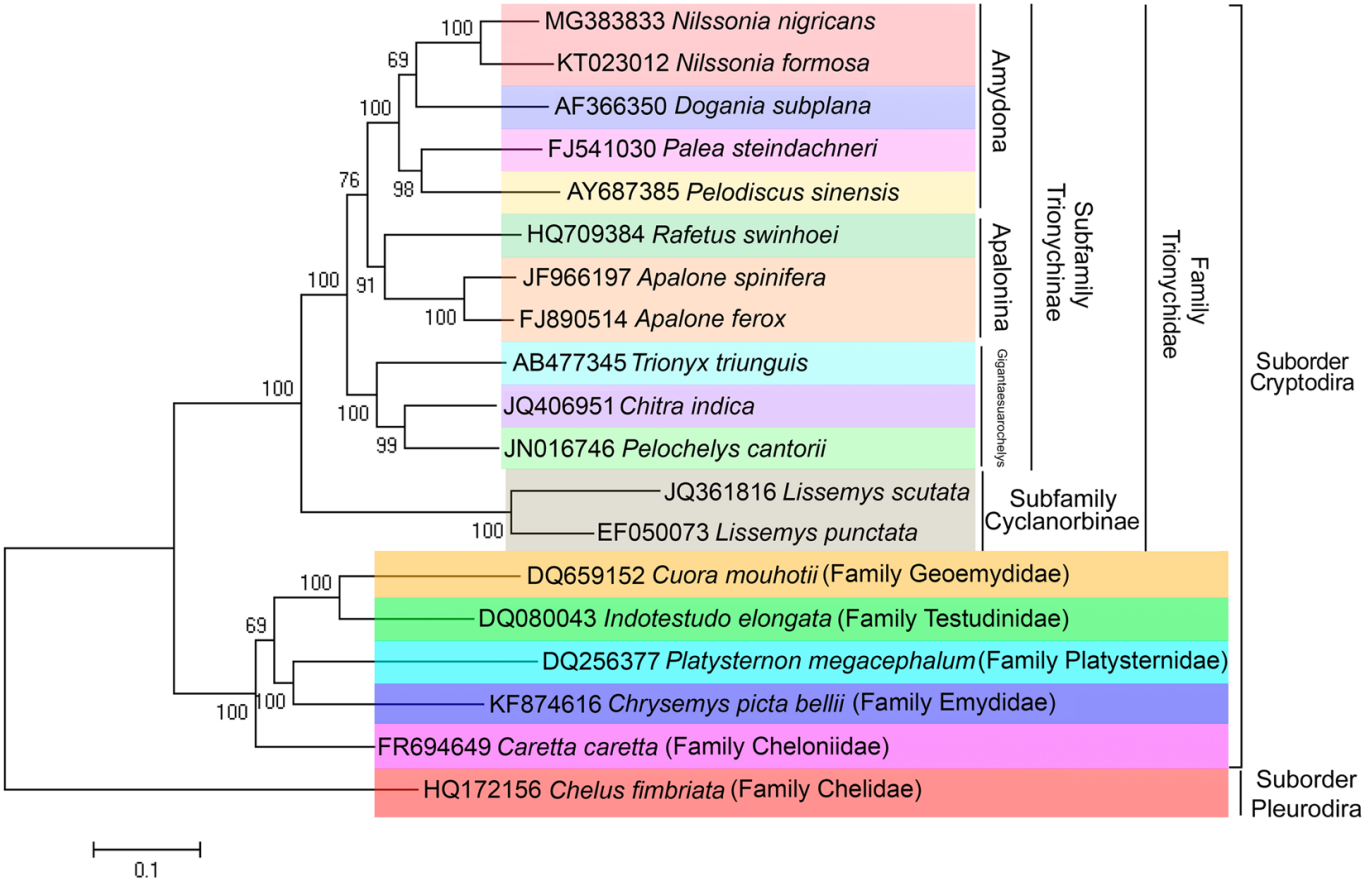
Maximum Likelihood phylogenetic tree based on the concatenated nucleotide sequences of 13 PCGs of the Trionychidae species showing the evolutionary relationship of *N. nigricans*. Bootstrap support values were superimposed with the nodes of ML tree. Species name and respective accession numbers were marked by different color bars as per clade pattern. Species specific taxonomic ranks (subfamily, family, and suborder) were incorporated with the specific clades. The figure was edited in Adobe Photoshop CS 8.0.

Phylogenetic relationships of the new *N. nigricans* mitogenome are shown relative to 19 species of Testudines from two suborders and seven families (Fig. 4). The phylogenetic tree revealed 13 species of Trionychidae family were clustered together with 100 percent bootstrap support and congruent with the previous phylogeny based hypothesis in Testudines systematics^2,3^. The current phylogeny places *N. nigricans* as sister group to *N. formosa* and supports the previous classification of Trionychidae species. The 11 species form three clades corresponding to tribes Amydona, Apalonina and Gigantaesuarochelys in the subfamily Trionychinae. The species of Geoemydidae and Testudinidae families clustered together and shows sister clade of Platysternidae and Emididae species (Fig. 4). The Cheloniidae species (sea turtle) joined the base of the ML tree. This phylogenetic analysis combined with all mitochondrial PCGs, is consistent with the previously described phylogeny using partial sequences of mitochondrial and nuclear genes^2,6,15,16,25^. However, further taxon sampling from different taxonomic ranks and their mitogenomics data would be useful for better understanding of the phylogenetic and evolutionary relationships among Testudines. In addition, the present study further confirmed the presence of *N. nigricans* in the tributaries of river Bramhaputra in Arunachal Pradesh state in northeast India. The previous and present locality information in wild habitats updated range distribution of *N. nigricans* in Bangaladesh and India (West Bengal, Assam, Tripura, Arunachal Pradesh).

## Electronic supplementary material

Supplementary Information
