# Supplementary material for: Complete mitochondrial genome of Black Soft-shell Turtle (Nilssonia nigricans) and comparative analysis with other Trionychidae
Source: Sci Rep. 2018 Nov 26;8:17378. doi: 10.1038/s41598-018-35822-5 (PMC6255766; doi:10.1038/s41598-018-35822-5)
Supplement: Supplementary file 1 — Supplementary Information [file 41598_2018_35822_MOESM1_ESM.docx]

**Supplementary Information**

**Complete mitochondrial genome of Black Soft-shell Turtle (*Nilssonia nigricans*) and comparative analysis with other Trionychidae**

Shantanu Kundu, Vikas Kumar, Kaomud Tyagi, Rajasree Chakraborty, Devkant Singha, Iftikar Rahaman, Avas Pakrashi, Kailash Chandra

*Centre for DNA Taxonomy*, *Molecular Systematics Division*, *Zoological Survey of India*, *M- Block*, *New Alipore*, *Kolkata- 700 053*, *West Bengal*, *India*

**Corresponding author’s Email:* [*vikaszsi77@gmail.com*](mailto:vikaszsi77@gmail.com)


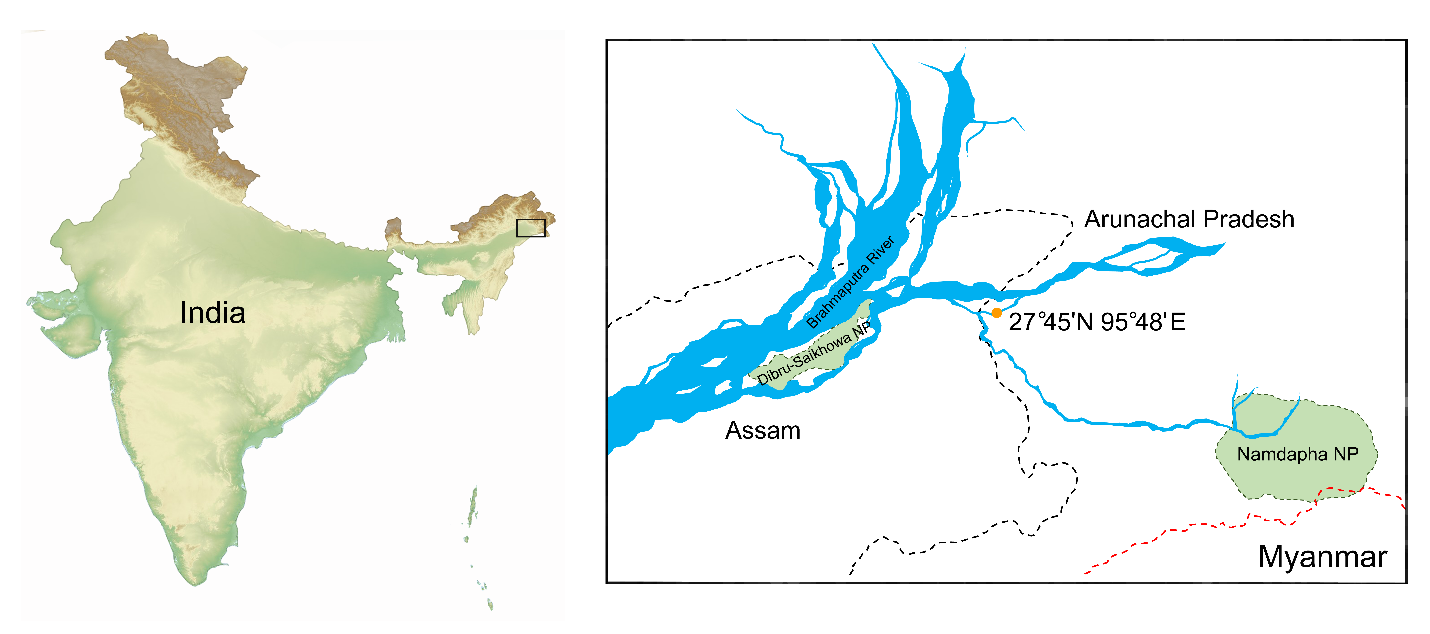
**Figure S1. The sampling site of *N. nigricans* in northeast India.** Map not to scale and manually prepared by the first author (S.K.) with the help of Google Map (<https://www.google.com/maps>) and Adobe Photoshop CS 8.0. The riverine systems are marked by blue color, national boundary between state Arunachal Pradesh and Assam is marked by Black dotted line, international boundary of India and Myanmar is marked by a Red dotted line, two National Parks are marked by Green colors, sampling sites with geographical coordinates is marked by Orange dot. The original template of the topographic map of India used here is copied under the following attribution: created by Yug (Own work) [CC BY-SA 3.0 http://creativecommons.org/licenses/by-sa/3.0)], via Wikimedia Commons; file URL: https://upload.wikimedia.org/wikipedia/commons/b/b9/Wikimaps_atlas-India-topographic_map-color-blank.jpg; page URL: https://commons.wikimedia.org/wiki/File%3AWikimaps_atlas-India-topographic_map-color-blank.jpg.


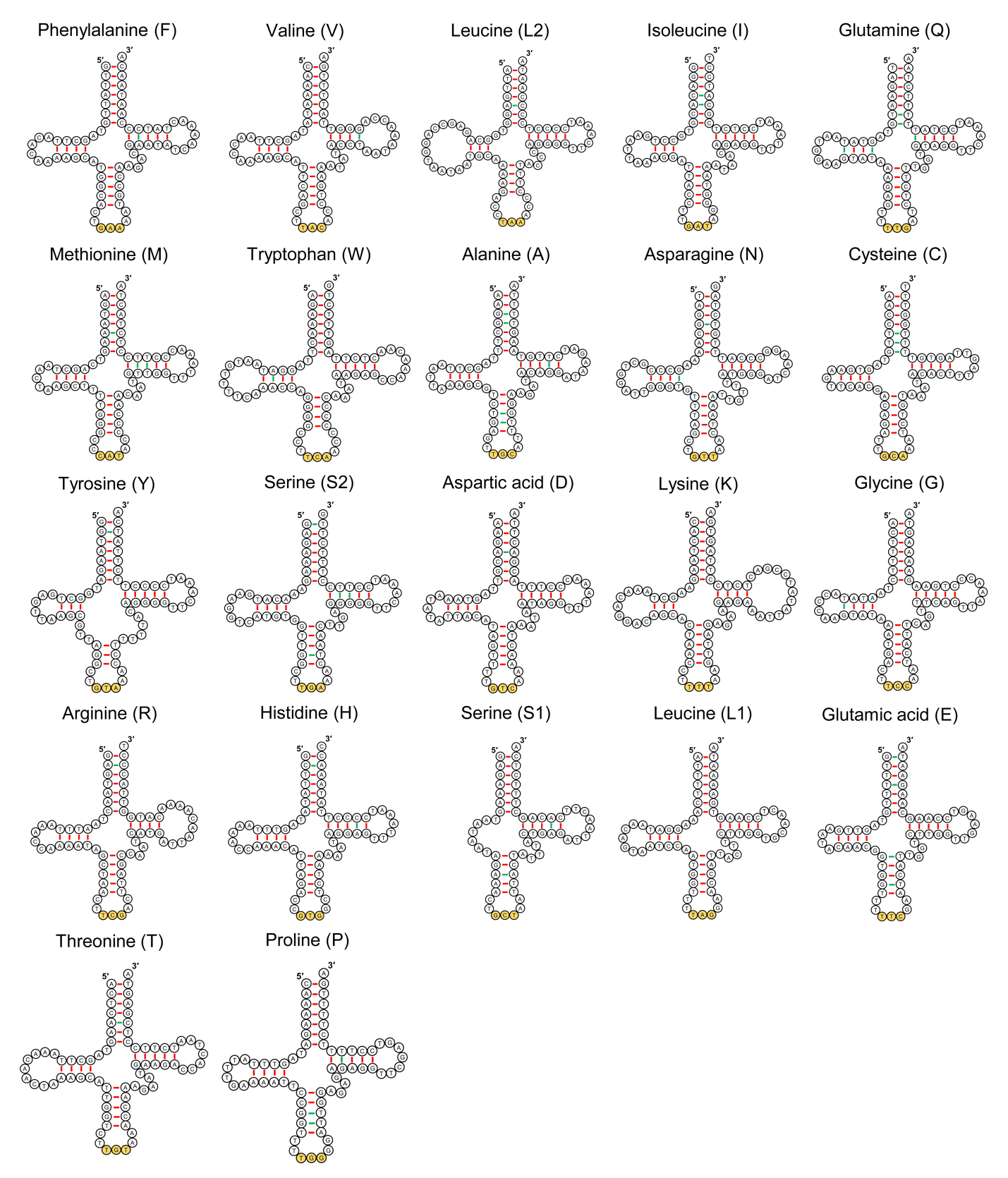
**Figure S2. Putative secondary structures for 22 tRNA genes in mitochondrial genome of *N. nigricans*.** The tRNAs are represented by full names and IUPAC-IUB single letter amino acid codes. Anticodons are marked by orange circles, red bars shows Watson-Crick base pairing, green bars shows wobble and mismatched base pairing. The secondary structure of tRNAs were predicted by the MITOS online server (http://mitos.bioinf.uni-leipzig.de/index.py) and edited manually in Adobe Photoshop CS 8.0.

**Table S1.** **Nucleotide composition of the mitochondrial genome in different Trionychidae species.** The A+T biases of whole mitogenomes, protein coding genes, tRNA, rRNA, and control regions were calculated by AT-skew = (A-T)/(A+T) and GC-skew= (G-C)/(G+C), respectively.

| **Species** | **Size (bp)** | **A%** | **T%** | **G%** | **C%** | **A+T%** | **AT- Skew** | **GC- Skew** |
| --- | --- | --- | --- | --- | --- | --- | --- | --- |
| **Complete mitogenome** | | | | | | | | |
| *A. ferox* | 16866 | 35.80 | 25.38 | 11.82 | 26.98 | 61.18 | 0.170 | -0.390 |
| *N. nigricans* | 16796 | 37.20 | 25.00 | 11.30 | 26.50 | 62.20 | 0.197 | -0.400 |
| *A. spinifera* | 16749 | 35.52 | 25.26 | 12.09 | 27.12 | 60.78 | 0.168 | -0.383 |
| *C. indica* | 16726 | 35.79 | 24.33 | 11.87 | 27.99 | 60.12 | 0.190 | -0.404 |
| *D. subplana* | 17289 | 35.29 | 25.96 | 11.94 | 26.78 | 61.26 | 0.152 | -0.383 |
| *L. punctate* | 16489 | 35.73 | 25.68 | 12.22 | 26.36 | 61.41 | 0.163 | -0.366 |
| *L. scutata* | 16512 | 35.97 | 25.41 | 12.04 | 26.56 | 61.38 | 0.172 | -0.376 |
| *N. Formosa* | 17145 | 37.13 | 25.83 | 11.19 | 25.82 | 62.97 | 0.179 | -0.395 |
| *P. steindachneri* | 17243 | 35.24 | 26.51 | 12.28 | 26.34 | 61.36 | 0.135 | -0.363 |
| *P. cantorii* | 17499 | 35.24 | 24.21 | 11.98 | 28.54 | 59.46 | 0.185 | -0.408 |
| *P. sinensis* | 17364 | 35.22 | 27.26 | 11.77 | 25.73 | 62.49 | 0.127 | -0.372 |
| *R. swinhoei* | 16990 | 34.98 | 25.46 | 12.24 | 27.30 | 60.45 | 0.157 | -0.380 |
| *T. triunguis* | 16590 | 35.34 | 23.14 | 12.20 | 29.31 | 58.48 | 0.208 | -0.412 |
| **Protein Coding genes (PCG)** | | | | | | | | |
| *A. ferox* | 11359 | 35.32 | 24.91 | 10.68 | 29.07 | 60.23 | 0.172 | -0.462 |
| *N. nigricans* | 11251 | 36.60 | 28.60 | 10.13 | 24.66 | 65.20 | 0.122 | -0.417 |
| *A. spinifera* | 11350 | 34.70 | 24.88 | 11.09 | 29.32 | 59.58 | 0.164 | -0.451 |
| *C. indica* | 11360 | 35.38 | 24.02 | 10.62 | 29.96 | 59.41 | 0.191 | -0.476 |
| *D. subplana* | 11335 | 35.13 | 25.77 | 10.82 | 28.26 | 60.90 | 0.153 | -0.446 |
| *L. punctate* | 11342 | 35.18 | 25.98 | 10.80 | 28.02 | 61.17 | 0.150 | -0.443 |
| *L. scutata* | 11356 | 35.37 | 25.73 | 10.68 | 28.20 | 61.11 | 0.157 | -0.450 |
| *N. Formosa* | 11357 | 36.98 | 25.12 | 10.05 | 27.84 | 62.10 | 0.190 | -0.469 |
| *P. steindachneri* | 11216 | 34.65 | 26.25 | 10.81 | 28.27 | 60.91 | 0.137 | -0.446 |
| *P. cantorii* | 11357 | 34.49 | 23.36 | 11.09 | 31.04 | 57.85 | 0.192 | -0.473 |
| *P. sinensis* | 11385 | 35.26 | 27.69 | 10.52 | 26.51 | 62.96 | 0.120 | -0.431 |
| *R. swinhoei* | 11384 | 34.59 | 25.14 | 11.09 | 29.16 | 59.74 | 0.158 | -0.448 |
| *T. triunguis* | 11352 | 34.39 | 22.87 | 11.17 | 31.55 | 57.26 | 0.201 | -0.476 |
| **tRNA genes** | | | | | | | | |
| *A. ferox* | 1602 | 35.95 | 27.65 | 14.29 | 22.09 | 63.60 | 0.130 | -0.214 |
| *N. nigricans* | 1551 | 36.49 | 27.33 | 14.18 | 21.98 | 63.82 | 0.140 | -0.220 |
| *A. spinifera* | 1594 | 35.57 | 27.41 | 14.74 | 22.27 | 62.98 | 0.129 | -0.203 |
| *C. indica* | 1601 | 35.47 | 26.92 | 14.61 | 22.98 | 62.39 | 0.137 | -0.222 |
| *D. subplana* | 1586 | 35.81 | 27.23 | 14.50 | 22.44 | 63.05 | 0.136 | -0.215 |
| *L. punctate* | 1600 | 35.37 | 26.68 | 14.87 | 23.06 | 62.06 | 0.139 | -0.215 |
| *L. scutata* | 1596 | 34.89 | 26.56 | 15.22 | 23.30 | 61.46 | 0.135 | -0.209 |
| *N. Formosa* | 1600 | 36.50 | 27.62 | 14.31 | 21.56 | 64.12 | 0.138 | -0.202 |
| *P. steindachneri* | 1589 | 35.30 | 27.69 | 15.10 | 21.90 | 62.99 | 0.120 | -0.183 |
| *P. cantorii* | 1591 | 34.63 | 25.89 | 15.27 | 24.19 | 60.52 | 0.144 | -0.226 |
| *P. sinensis* | 1617 | 35.37 | 28.57 | 14.34 | 21.70 | 63.94 | 0.106 | -0.204 |
| *R. swinhoei* | 1586 | 35.18 | 27.23 | 14.81 | 22.76 | 62.42 | 0.127 | -0.211 |
| *T. triunguis* | 1587 | 35.47 | 26.08 | 14.87 | 23.56 | 61.56 | 0.152 | -0.226 |
| **rRNA genes** | | | | | | | | |
| *A. ferox* | 2584 | 40.05 | 21.20 | 15.86 | 22.87 | 61.26 | 0.307 | -0.180 |
| *N. nigricans* | 2593 | 40.18 | 21.28 | 15.77 | 22.75 | 61.47 | 0.307 | -0.181 |
| *A. spinifera* | 2585 | 40.11 | 21.04 | 16.05 | 22.78 | 61.16 | 0.311 | -0.173 |
| *C. indica* | 2570 | 39.37 | 20.38 | 16.53 | 23.69 | 59.76 | 0.317 | -0.177 |
| *D. subplana* | 2578 | 39.13 | 21.72 | 16.13 | 23.00 | 60.86 | 0.286 | -0.175 |
| *L. punctate* | 2547 | 38.43 | 21.94 | 16.92 | 22.69 | 60.38 | 0.273 | -0.145 |
| *L. scutata* | 2585 | 39.38 | 21.47 | 16.28 | 22.86 | 60.85 | 0.294 | -0.167 |
| *N. Formosa* | 2594 | 40.28 | 21.81 | 15.57 | 22.32 | 62.10 | 0.297 | -0.178 |
| *P. steindachneri* | 2585 | 39.30 | 21.27 | 16.20 | 23.21 | 60.58 | 0.297 | -0.177 |
| *P. cantorii* | 2567 | 39.65 | 20.17 | 16.40 | 23.76 | 59.83 | 0.325 | -0.183 |
| *P. sinensis* | 2687 | 39.18 | 22.55 | 15.96 | 22.29 | 61.74 | 0.269 | -0.165 |
| *R. swinhoei* | 2573 | 38.63 | 21.33 | 16.75 | 23.28 | 59.96 | 0.288 | -0.163 |
| *T. triunguis* | 2586 | 39.24 | 19.76 | 16.04 | 24.94 | 59.01 | 0.330 | -0.216 |
| **Control regions (CR)** | | | | | | | | |
| *A. ferox* | 1357 | 31.61 | 35.74 | 10.31 | 22.32 | 67.35 | -0.061 | -0.367 |
| *N. nigricans* | 1290 | 36.66 | 31.86 | 9.84 | 21.62 | 68.52 | 0.070 | -0.374 |
| *A. spinifera* | 1234 | 33.22 | 36.14 | 9.23 | 21.39 | 69.36 | -0.042 | -0.396 |
| *C. indica* | 1227 | 33.00 | 32.92 | 10.10 | 23.96 | 65.93 | 0.001 | -0.406 |
| *D. subplana* | 1820 | 30.54 | 32.91 | 10.60 | 25.93 | 63.46 | -0.037 | -0.419 |
| *L. punctate* | 1008 | 34.92 | 31.74 | 11.80 | 21.52 | 66.66 | 0.047 | -0.291 |
| *L. scutata* | 1005 | 35.22 | 31.24 | 11.64 | 21.89 | 66.46 | 0.059 | -0.305 |
| *N. Formosa* | 1604 | 33.97 | 36.34 | 8.54 | 21.13 | 70.32 | -0.033 | -0.424 |
| *P. steindachneri* | 1736 | 29.37 | 36.34 | 13.19 | 21.08 | 65.72 | -0.106 | -0.230 |
| *P. cantorii* | 1994 | 34.65 | 33.75 | 8.62 | 22.96 | 68.40 | 0.0130 | -0.453 |
| *P. sinensis* | 1830 | 29.50 | 31.03 | 10.87 | 28.57 | 60.54 | -0.025 | -0.448 |
| *R. swinhoei* | 1471 | 31.67 | 34.46 | 10.12 | 23.72 | 66.14 | -0.042 | -0.401 |
| *T. triunguis* | 1078 | 35.80 | 31.26 | 9.18 | 23.74 | 67.06 | 0.067 | -0.442 |

**Table S2. Comparison of gene rearrangements in light and heavy strand within 13 Trionychidae species.** *nn= N. nigricans, nf= N. formosa, af= A. ferox, as= A. spinifera, ci= C. indica, ds= D. subplana, lp= L. punctata, lc= L. scutata, pst= P. steindachneri, pc= P. cantorii, psi= P. sinensis, rs= R. swinhoei, tt= T. triunguis.* tRNAs are encoded according to their single-letter abbreviations.

| **Species** | F | rrnS | V | rrnL | L2 | nad1 | I | Q | M | nad2 | W | A | N | C | Y | cox1 | S2 | D | cox2 | K | atp8 | atp6 | cox3 | G | nad3 | R | nad4l | nad4 | H | S1 | L1 | nad5 | nad6 | E | cytb | T | P | CR |
| --- | --- | --- | --- | --- | --- | --- | --- | --- | --- | --- | --- | --- | --- | --- | --- | --- | --- | --- | --- | --- | --- | --- | --- | --- | --- | --- | --- | --- | --- | --- | --- | --- | --- | --- | --- | --- | --- | --- |
| ***nn*** | + | + | + | + | + | + | + | - | + | + | + | - | - | - | - | + | - | + | + | + | + | + | + | + | + | + | + | + | + | + | + | + | - | - | + | + | - | . |
| ***nf*** | + | + | + | + | + | + | + | - | + | + | + | - | - | - | - | + | - | + | + | + | + | + | + | + | + | + | + | + | + | + | + | + | - | - | + | + | - | . |
| ***ds*** | + | + | + | + | + | + | + | - | + | + | + | - | - | - | - | + | - | + | + | + | + | + | + | + | + | + | + | + | + | + | + | + | - | - | + | + | - | . |
| ***pst*** | + | + | + | + | + | + | + | - | + | + | + | - | - | - | - | + | - | + | + | + | + | + | + | + | + | + | + | + | + | + | + | + | - | - | + | + | - | . |
| ***psi*** | + | + | + | + | + | + | + | - | + | + | + | - | - | - | - | + | - | + | + | + | + | + | + | + | + | + | + | + | + | + | + | + | - | - | + | + | - | . |
| ***rs*** | + | + | + | + | + | + | + | - | + | + | + | - | - | - | - | + | - | + | + | + | + | + | + | + | + | + | + | + | + | + | + | + | - | - | + | + | - | . |
| ***as*** | + | + | + | + | + | + | + | - | + | + | + | - | - | - | - | + | - | + | + | + | + | + | + | + | + | + | + | + | + | + | + | + | - | - | + | + | - | . |
| ***af*** | + | + | + | + | + | + | + | - | + | + | + | - | - | - | - | + | - | + | + | + | + | + | + | + | + | + | + | + | + | + | + | + | - | - | + | + | - | . |
| ***tt*** | + | + | + | + | + | + | + | - | + | + | + | - | - | - | - | + | - | + | + | + | + | + | + | + | + | + | + | + | + | + | + | + | - | - | + | + | - | . |
| ***ci*** | + | + | + | + | + | + | + | - | + | + | + | - | - | - | - | + | - | + | + | + | + | + | + | + | + | + | + | + | + | + | + | + | - | - | + | + | - | . |
| ***pc*** | + | + | + | + | + | + | + | - | + | + | + | - | - | - | - | + | - | + | + | + | + | + | + | + | + | + | + | + | + | + | + | + | - | - | + | + | - | . |
| ***ls*** | + | + | + | + | + | + | + | - | + | + | + | - | - | - | - | + | - | + | + | + | + | + | + | + | + | + | + | + | + | + | + | + | - | - | + | + | - | . |
| ***lp*** | + | + | + | + | + | + | + | - | + | + | + | - | - | - | - | + | - | + | + | + | + | + | + | + | + | + | + | + | + | + | + | + | - | - | + | + | - | . |

**Table S3. Comparison of overlapping and intergenic spacer regions of 13 Trionychidae species in the current study.** *nn= N. nigricans, nf= N. formosa, af= A. ferox, as= A. spinifera, ci= C. indica, ds= D. subplana, lp= L. punctata, lc= L. scutata, pst= P. steindachneri, pc= P. cantorii, psi= P. sinensis, rs= R. swinhoei, tt= T. triunguis.*

| **Locus** | *nn* | *nf* | *ds* | *pst* | *psi* | *rs* | *as* | *af* | *tt* | *ci* | *Pc* | *ls* | *lp* |
| --- | --- | --- | --- | --- | --- | --- | --- | --- | --- | --- | --- | --- | --- |
| *trnF* | 0 | 0 | 0 | 0 | 0 | 0 | 0 | 0 | 0 | 0 | 0 | 0 | 0 |
| *rrnS* | -2 | -2 | 0 | 0 | -100 | 0 | 0 | 0 | 0 | 0 | 0 | 0 | 0 |
| *trnV* | 0 | 0 | 0 | -2 | 0 | 0 | 0 | 0 | 0 | 0 | 0 | 0 | 0 |
| *rrnL* | 0 | 0 | 0 | 0 | 0 | 0 | 0 | 0 | 0 | 0 | 0 | 0 | 6 |
| *trnL2* | 13 | 0 | 1 | -3 | 0 | 0 | 0 | 0 | 0 | 0 | 0 | 0 | 0 |
| *nad1* | 8 | 0 | -1 | 0 | -1 | 0 | 0 | 0 | 0 | 0 | 1 | 0 | 0 |
| *trnI* | -1 | -2 | -1 | -1 | -1 | -1 | -1 | -1 | -1 | -1 | -1 | -1 | -1 |
| *trnQ* | -1 | -1 | -1 | -1 | 4 | -1 | -1 | -1 | -1 | 3 | 4 | -1 | -1 |
| *trnM* | 0 | 0 | 0 | 0 | 0 | 0 | 0 | 0 | 0 | 0 | 0 | 0 | 0 |
| *nad2* | 13 | 0 | -2 | 0 | -2 | 0 | 0 | 0 | 0 | 0 | 0 | 0 | 0 |
| *trnW* | 4 | 4 | 9 | 8 | 0 | 12 | 13 | 3 | 11 | 10 | 8 | 4 | 3 |
| *trnA* | 1 | 1 | 1 | 1 | 1 | 1 | 1 | 1 | 1 | 1 | 1 | 1 | 1 |
| *trnN* | 33 | 32 | 29 | 29 | 31 | 31 | 30 | 31 | 31 | 30 | 30 | 33 | 33 |
| *trnC* | 0 | 0 | 0 | 0 | -2 | 0 | 0 | 0 | 0 | -3 | 0 | 0 | -1 |
| *trnY* | 4 | 3 | 1 | 1 | 1 | 1 | 1 | 1 | 1 | 1 | 1 | 1 | 1 |
| *cox1* | 4 | 0 | 5 | -5 | -5 | -5 | -5 | -5 | 2 | -9 | -5 | -9 | 7 |
| *trnS2* | 0 | 0 | 0 | 0 | 1 | 0 | 0 | 0 | 0 | 0 | 0 | 0 | 0 |
| *trnD* | 0 | 0 | 0 | 0 | 0 | 0 | 0 | 0 | 0 | 0 | 0 | 0 | 0 |
| *cox2* | 7 | 1 | 1 | 1 | 1 | 1 | 1 | 1 | 1 | 2 | 1 | 1 | 2 |
| *trnK* | 1 | 0 | 1 | 1 | 1 | 1 | 1 | 1 | 1 | 2 | 1 | 1 | 2 |
| *atp8* | -4 | 0 | -10 | -10 | -10 | -10 | -10 | -10 | -10 | -10 | -10 | -10 | -10 |
| *atp6* | 2 | 0 | -1 | -1 | -1 | -1 | -1 | -1 | -1 | -1 | -1 | -1 | 0 |
| *cox3* | 1 | 0 | 0 | 3 | 1 | 0 | 0 | 0 | 0 | 0 | 0 | 0 | 0 |
| *trnG* | 0 | 0 | 0 | 0 | 0 | 0 | 0 | 0 | 0 | 0 | 0 | 0 | 0 |
| *nad3* | 1 | 0 | 0 | 0 | -1 | 0 | 0 | 0 | 0 | 0 | 0 | 0 | 0 |
| *trnR* | 1 | 0 | 1 | 0 | 0 | 0 | 0 | 0 | 0 | 0 | 0 | 0 | 0 |
| *nad4l* | -4 | 0 | -5 | -7 | -7 | -6 | -7 | -7 | -7 | -7 | -7 | -7 | -7 |
| *nad4* | 10 | 0 | 0 | -3 | -5 | -2 | 0 | 0 | 0 | 0 | 0 | 0 | 0 |
| *trnH* | 0 | 0 | 0 | 0 | 0 | 0 | 0 | 0 | 0 | 0 | 0 | 0 | 0 |
| *trnS1* | -1 | -1 | -1 | 0 | -1 | -1 | -1 | -1 | -1 | -1 | -1 | -1 | -1 |
| *trnL1* | 0 | 0 | 0 | 0 | 0 | 0 | 0 | 0 | 0 | 0 | 0 | 0 | 0 |
| *nad5* | 1 | 0 | -5 | -5 | -5 | -5 | -5 | -5 | -5 | -5 | -5 | -8 | -5 |
| *nad6* | 0 | 2 | -3 | -3 | 0 | 0 | -3 | -3 | 0 | -3 | 0 | 0 | 0 |
| *trnE* | 9 | 6 | 3 | 3 | -2 | 3 | 3 | 3 | 3 | 3 | 3 | 4 | 3 |
| *cytb* | 9 | -1 | 2 | 3 | 3 | 0 | 14 | 0 | 3 | 3 | 3 | 6 | 6 |
| *trnT* | 2 | 2 | 3 | 19 | 1 | 14 | 12 | 12 | 15 | 9 | 23 | 13 | 10 |
| *trnP* | 0 | 0 | 0 | 0 | 0 | 0 | 0 | 0 | 0 | 0 | 0 | 0 | 0 |
| A+T-rich Region | - | - | - | - | - | - |  | - | - | - | - | - | - |

**Table S4. Comparison of start and stop codons of PCGs within 13 Trionychidae species.** *nn= N. nigricans, nf= N. formosa, af= A. ferox, as= A. spinifera, ci= C. indica, ds= D. subplana, lp= L. punctata, lc= L. scutata, pst= P. steindachneri, pc= P. cantorii, psi= P. sinensis, rs= R. swinhoei, tt= T. triunguis.*

| **Locus** | ***nn*** | | ***nf*** | | ***ds*** | | ***pst*** | | ***psi*** | | ***rs*** | | ***as*** | | ***af*** | | ***tt*** | | ***ci*** | | ***Pc*** | | ***ls*** | | ***lp*** | |
| --- | --- | --- | --- | --- | --- | --- | --- | --- | --- | --- | --- | --- | --- | --- | --- | --- | --- | --- | --- | --- | --- | --- | --- | --- | --- | --- |
|  | Start | Stop | Start | Stop | Start | Stop | Start | Stop | Start | Stop | Start | Stop | Start | Stop | Start | Stop | Start | Stop | Start | Stop | Start | Stop | Start | Stop | Start | Stop |
| *nad1* | ATA | A | ATG | T(AA) | ATG | TAG | ATA | TA(A) | ATG | TAG | ATG | TA(A) | ATG | TA(A) | ATG | TA(A) | ATG | TAA | ATG | TAA | ATG | TAA | ATG | TA(A) | ATG | TAG |
| *nad2* | ATG | A | ATG | TAA | ATG | TAG | ATG | T(AA) | ATG | TAG | ATG | TA(A) | ATG | T(AA) | ATG | T(AA) | ATG | T(AA) | ATG | T(AA) | ATG | T(AA) | ATG | T(AA) | ATG | T(AA) |
| *cox1* | ATT | A | ATT | AGA | GTG | TAA | GTG | AGA | GTG | AGA | GTG | AGA | GTG | AGA | GTG | AGA | GTG | TAA | GTG | AGG | GTG | AGA | GTG | AGA | GTG | TAA |
| *cox2* | ATG | T | ATG | TAA | ATG | TAA | ATG | TAA | ATG | TAA | ATG | TAA | ATG | TAA | ATG | TAA | ATG | TAA | ATG | TAA | ATG | TAA | ATG | TAA | ATG | TAA |
| *atp8* | ATG | A | ATG | TAA | ATG | TAA | ATG | TAA | ATG | TAA | ATG | TAA | ATG | TAA | ATG | TAA | ATG | TAA | ATG | TAA | ATG | TAA | ATG | TAA | ATG | TAA |
| *atp6* | ATG | (TAA) | ATG | TAA | ATG | TAA | ATG | TAA | ATG | TAA | ATG | TAA | ATG | TAA | ATG | TAA | ATG | TAA | ATG | TAA | ATG | TAA | ATG | TAA | ATG | TAA |
| *cox3* | ATG | T | ATG | T(AA) | ATG | T(AA) | ATG | T(AA) | ATG | TAA | ATG | T(AA) | ATG | T(AA) | ATG | T(AA) | ATG | T(AA) | ATG | T(AA) | ATG | T(AA) | ATG | T(AA) | ATG | T(AA) |
| *nad3* | ATG | AGA | ATG | T(AA) | ATG | T(AA) | ATG | T(AA) | ATG | TAA | ATG | T(AA) | ATG | T(AA) | ATG | T(AA) | ATG | AGA | ATG | T(AA) | ATG | T(AA) | ATG | T(AA) | ATG | T(AA) |
| *nad4l* | ATG | (TAA) | ATG | TAA | ATG | T(AA) | ATG | TAA | ATG | TAA | ATG | TA(A) | ATG | TAA | ATG | TAA | ATG | TAA | ATG | TAA | ATG | TAA | ATG | TAA | ATG | TAA |
| *nad4* | ATG | C | ATG | TAA | ATG | T(AA) | ATG | T(AA) | ATG | TAA | ATG | T(AA) | ATG | T(AA) | ATG | T(AA) | ATG | T(AA) | ATG | T(AA) | ATG | T(AA) | ATG | T(AA) | ATG | T(AA) |
| *nad5* | ATG | (TAA) | ATG | TAA | ATG | TAA | ATG | TAA | ATG | TAA | ATG | TAA | ATG | TAA | ATG | TAA | ATG | TAA | ATG | TAA | ATG | TAA | GTG | TAG | ATG | TAA |
| *nad6* | ATG | T | ATG | AGA | ATG | AGA | ATA | AGA | ATG | AGG | ATG | AGA | ATA | AGA | ATA | AGA | ATG | AGA | ATA | AGA | ATG | AGA | ATG | AGA | ATG | AGA |
| *cytb* | ATC | A | ATC | A | ATG | TAA | ATG | TAA | ATT | TAA | ATG | T(AA) | ATG | TAA | ATG | T(AA) | ATG | TAA | ATG | TAA | ATG | TAA | ATG | TAA | ATG | TAA |

**Table S5. The RSCU value of the complete PCGs of compares 13 Trionychidae species.**

| ***N. nigricans*** | | | | | | | | | | | | | | |
| --- | --- | --- | --- | --- | --- | --- | --- | --- | --- | --- | --- | --- | --- | --- |
| Codon | | RSCU | | Codon | RSCU | Codon | | | | RSCU | Codon | | RSCU | |
| UUU(F) | | 0.93 | | UCU(S) | 0.6 | UAU(Y) | | | | 0.93 | UGU(C) | | 1.14 | |
| UUC(F) | | 1.07 | | UCC(S) | 0.82 | UAC(Y) | | | | 1.07 | UGC(C) | | 0.86 | |
| UUA(L) | | 1.26 | | UCA(S) | 2.13 | UAA(*) | | | | 0.4 | UGA(W) | | 1.38 | |
| UUG(L) | | 0.15 | | UCG(S) | 0.15 | UAG(*) | | | | 0.32 | UGG(W) | | 0.62 | |
| CUU(L) | | 0.84 | | CCU(P) | 1.63 | CAU(H) | | | | 1.07 | CGU(R) | | 0.47 | |
| CUC(L) | | 0.76 | | CCC(P) | 0.64 | CAC(H) | | | | 0.93 | CGC(R) | | 1.19 | |
| CUA(L) | | 2.73 | | CCA(P) | 1.57 | CAA(Q) | | | | 1.85 | CGA(R) | | 1.7 | |
| CUG(L) | | 0.27 | | CCG(P) | 0.17 | CAG(Q) | | | | 0.15 | CGG(R) | | 0.64 | |
| AUU(I) | | 1.02 | | ACU(T) | 1.22 | AAU(N) | | | | 0.97 | AGU(S) | | 0.63 | |
| AUC(I) | | 0.98 | | ACC(T) | 1.05 | AAC(N) | | | | 1.03 | AGC(S) | | 1.67 | |
| AUA(M) | | 1.55 | | ACA(T) | 1.56 | AAA(K) | | | | 1.83 | AGA(*) | | 1.24 | |
| AUG(M) | | 0.45 | | ACG(T) | 0.17 | AAG(K) | | | | 0.17 | AGG(*) | | 2.04 | |
| GUU(V) | | 1 | | GCU(A) | 0.6 | GAU(D) | | | | 0.4 | GGU(G) | | 1 | |
| GUC(V) | | 0.7 | | GCC(A) | 1.7 | GAC(D) | | | | 1.6 | GGC(G) | | 1.06 | |
| GUA(V) | | 2.19 | | GCA(A) | 1.67 | GAA(E) | | | | 1.77 | GGA(G) | | 1.55 | |
| GUG(V) | | 0.11 | | GCG(A) | 0.03 | GAG(E) | | | | 0.23 | GGG(G) | | 0.39 | |
| ***N. Formosa*** | | | | | | | | | | | | | | |
| Codon | | RSCU | | Codon | RSCU | Codon | | | | RSCU | Codon | | RSCU | |
| UUU(F) | | 1.09 | | UCU(S) | 0.89 | UAU(Y) | | | | 1.12 | UGU(C) | | 1.26 | |
| UUC(F) | | 0.91 | | UCC(S) | 0.98 | UAC(Y) | | | | 0.88 | UGC(C) | | 0.74 | |
| UUA(L) | | 1.16 | | UCA(S) | 1.29 | UAA(*) | | | | 1.4 | UGA(W) | | 0.87 | |
| UUG(L) | | 0.43 | | UCG(S) | 0.38 | UAG(*) | | | | 0.64 | UGG(W) | | 1.13 | |
| CUU(L) | | 1.46 | | CCU(P) | 1.38 | CAU(H) | | | | 1.05 | CGU(R) | | 0.92 | |
| CUC(L) | | 0.96 | | CCC(P) | 0.89 | CAC(H) | | | | 0.95 | CGC(R) | | 0.96 | |
| CUA(L) | | 1.41 | | CCA(P) | 1.46 | CAA(Q) | | | | 1.64 | CGA(R) | | 1.08 | |
| CUG(L) | | 0.59 | | CCG(P) | 0.27 | CAG(Q) | | | | 0.36 | CGG(R) | | 1.04 | |
| AUU(I) | | 1.22 | | ACU(T) | 1.67 | AAU(N) | | | | 1.14 | AGU(S) | | 0.78 | |
| AUC(I) | | 0.78 | | ACC(T) | 0.97 | AAC(N) | | | | 0.86 | AGC(S) | | 1.66 | |
| AUA(M) | | 1.28 | | ACA(T) | 1.07 | AAA(K) | | | | 1.66 | AGA(*) | | 0.79 | |
| AUG(M) | | 0.72 | | ACG(T) | 0.3 | AAG(K) | | | | 0.34 | AGG(*) | | 1.17 | |
| GUU(V) | | 1.65 | | GCU(A) | 0.77 | GAU(D) | | | | 1.24 | GGU(G) | | 1.67 | |
| GUC(V) | | 0.96 | | GCC(A) | 2.14 | GAC(D) | | | | 0.76 | GGC(G) | | 0.83 | |
| GUA(V) | | 0.87 | | GCA(A) | 0.88 | GAA(E) | | | | 1.38 | GGA(G) | | 0.67 | |
| GUG(V) | | 0.52 | | GCG(A) | 0.22 | GAG(E) | | | | 0.63 | GGG(G) | | 0.83 | |
| ***A. ferox*** | | | | | | | | | | | | | | |
| Codon | | RSCU | | Codon | RSCU | Codon | | | | RSCU | Codon | | RSCU | |
| UUU(F) | | 0.75 | | UCU(S) | 0.95 | UAU(Y) | | | | 0.82 | UGU(C) | | 0.64 | |
| UUC(F) | | 1.25 | | UCC(S) | 1.27 | UAC(Y) | | | | 1.18 | UGC(C) | | 1.36 | |
| UUA(L) | | 1.57 | | UCA(S) | 2.76 | UAA(*) | | | | 2.77 | UGA(W) | | 1.71 | |
| UUG(L) | | 0.34 | | UCG(S) | 0.29 | UAG(*) | | | | 1.02 | UGG(W) | | 0.29 | |
| CUU(L) | | 0.58 | | CCU(P) | 0.79 | CAU(H) | | | | 0.81 | CGU(R) | | 0.4 | |
| CUC(L) | | 0.59 | | CCC(P) | 0.84 | CAC(H) | | | | 1.19 | CGC(R) | | 0.73 | |
| CUA(L) | | 2.63 | | CCA(P) | 2.18 | CAA(Q) | | | | 1.76 | CGA(R) | | 2.8 | |
| CUG(L) | | 0.28 | | CCG(P) | 0.19 | CAG(Q) | | | | 0.24 | CGG(R) | | 0.07 | |
| AUU(I) | | 0.8 | | ACU(T) | 0.75 | AAU(N) | | | | 0.53 | AGU(S) | | 0.17 | |
| AUC(I) | | 1.2 | | ACC(T) | 1.32 | AAC(N) | | | | 1.47 | AGC(S) | | 0.56 | |
| AUA(M) | | 1.67 | | ACA(T) | 1.81 | AAA(K) | | | | 1.79 | AGA(*) | | 0.15 | |
| AUG(M) | | 0.33 | | ACG(T) | 0.12 | AAG(K) | | | | 0.21 | AGG(*) | | 0.06 | |
| GUU(V) | | 0.91 | | GCU(A) | 0.68 | GAU(D) | | | | 0.74 | GGU(G) | | 0.71 | |
| GUC(V) | | 0.52 | | GCC(A) | 1.91 | GAC(D) | | | | 1.26 | GGC(G) | | 1.01 | |
| GUA(V) | | 2.07 | | GCA(A) | 1.33 | GAA(E) | | | | 1.66 | GGA(G) | | 1.65 | |
| GUG(V) | | 0.5 | | GCG(A) | 0.08 | GAG(E) | | | | 0.34 | GGG(G) | | 0.64 | |
| ***A. spinifera*** | | | | | | | | | | | | | | |
| Codon | | RSCU | | Codon | RSCU | Codon | | | | RSCU | Codon | | RSCU | |
| UUU(F) | | 0.79 | | UCU(S) | 0.65 | UAU(Y) | | | | 1.05 | UGU(C) | | 1.29 | |
| UUC(F) | | 1.21 | | UCC(S) | 1.08 | UAC(Y) | | | | 0.95 | UGC(C) | | 0.71 | |
| UUA(L) | | 1.37 | | UCA(S) | 2.2 | UAA(*) | | | | 1.86 | UGA(W) | | 1.38 | |
| UUG(L) | | 0.25 | | UCG(S) | 0.37 | UAG(*) | | | | 0.67 | UGG(W) | | 0.62 | |
| CUU(L) | | 0.67 | | CCU(P) | 1.18 | CAU(H) | | | | 0.92 | CGU(R) | | 0.63 | |
| CUC(L) | | 0.75 | | CCC(P) | 0.98 | CAC(H) | | | | 1.08 | CGC(R) | | 1.03 | |
| CUA(L) | | 2.56 | | CCA(P) | 1.67 | CAA(Q) | | | | 1.76 | CGA(R) | | 1.84 | |
| CUG(L) | | 0.39 | | CCG(P) | 0.18 | CAG(Q) | | | | 0.24 | CGG(R) | | 0.49 | |
| AUU(I) | | 0.8 | | ACU(T) | 1.01 | AAU(N) | | | | 0.9 | AGU(S) | | 0.41 | |
| AUC(I) | | 1.2 | | ACC(T) | 1.24 | AAC(N) | | | | 1.1 | AGC(S) | | 1.29 | |
| AUA(M) | | 1.55 | | ACA(T) | 1.62 | AAA(K) | | | | 1.77 | AGA(*) | | 0.54 | |
| AUG(M) | | 0.45 | | ACG(T) | 0.13 | AAG(K) | | | | 0.23 | AGG(*) | | 0.93 | |
| GUU(V) | | 1.16 | | GCU(A) | 0.77 | GAU(D) | | | | 0.67 | GGU(G) | | 0.83 | |
| GUC(V) | | 0.67 | | GCC(A) | 1.7 | GAC(D) | | | | 1.33 | GGC(G) | | 1.14 | |
| GUA(V) | | 1.77 | | GCA(A) | 1.51 | GAA(E) | | | | 1.79 | GGA(G) | | 1.37 | |
| GUG(V) | | 0.4 | | GCG(A) | 0.03 | GAG(E) | | | | 0.21 | GGG(G) | | 0.67 | |
| ***C. indica*** | | | | | | | | | | | | | | |
| Codon | | RSCU | | Codon | RSCU | Codon | | | | RSCU | Codon | | RSCU | |
| UUU(F) | | 1.05 | | UCU(S) | 1.05 | UAU(Y) | | | | 0.95 | UGU(C) | | 1.21 | |
| UUC(F) | | 0.95 | | UCC(S) | 1.25 | UAC(Y) | | | | 1.05 | UGC(C) | | 0.79 | |
| UUA(L) | | 0.91 | | UCA(S) | 1.57 | UAA(*) | | | | 1.4 | UGA(W) | | 0.93 | |
| UUG(L) | | 0.68 | | UCG(S) | 0.34 | UAG(*) | | | | 0.67 | UGG(W) | | 1.07 | |
| CUU(L) | | 1.02 | | CCU(P) | 1.17 | CAU(H) | | | | 1.11 | CGU(R) | | 1.03 | |
| CUC(L) | | 1.06 | | CCC(P) | 0.92 | CAC(H) | | | | 0.89 | CGC(R) | | 1.03 | |
| CUA(L) | | 1.78 | | CCA(P) | 1.65 | CAA(Q) | | | | 1.42 | CGA(R) | | 0.95 | |
| CUG(L) | | 0.55 | | CCG(P) | 0.26 | CAG(Q) | | | | 0.58 | CGG(R) | | 0.99 | |
| AUU(I) | | 1.15 | | ACU(T) | 1.47 | AAU(N) | | | | 1.09 | AGU(S) | | 0.61 | |
| AUC(I) | | 0.85 | | ACC(T) | 0.94 | AAC(N) | | | | 0.91 | AGC(S) | | 1.18 | |
| AUA(M) | | 1.17 | | ACA(T) | 1.25 | AAA(K) | | | | 1.68 | AGA(*) | | 0.98 | |
| AUG(M) | | 0.83 | | ACG(T) | 0.33 | AAG(K) | | | | 0.32 | AGG(*) | | 0.95 | |
| GUU(V) | | 1.48 | | GCU(A) | 0.61 | GAU(D) | | | | 1.02 | GGU(G) | | 0.8 | |
| GUC(V) | | 0.61 | | GCC(A) | 1.86 | GAC(D) | | | | 0.98 | GGC(G) | | 1.17 | |
| GUA(V) | | 0.96 | | GCA(A) | 1.25 | GAA(E) | | | | 1.34 | GGA(G) | | 1.05 | |
| GUG(V) | | 0.96 | | GCG(A) | 0.28 | GAG(E) | | | | 0.66 | GGG(G) | | 0.98 | |
| ***D. subplana*** | | | | | | | | | | | | | | |
| Codon | RSCU | | | Codon | RSCU | Codon | | | | RSCU | Codon | | RSCU | |
| UUU(F) | 0.9 | | | UCU(S) | 0.65 | UAU(Y) | | | | 1.04 | UGU(C) | | 1.16 | |
| UUC(F) | 1.1 | | | UCC(S) | 0.9 | UAC(Y) | | | | 0.96 | UGC(C) | | 0.84 | |
| UUA(L) | 1.56 | | | UCA(S) | 2.26 | UAA(*) | | | | 1 | UGA(W) | | 1.47 | |
| UUG(L) | 0.16 | | | UCG(S) | 0.12 | UAG(*) | | | | 0.52 | UGG(W) | | 0.53 | |
| CUU(L) | 0.86 | | | CCU(P) | 1.15 | CAU(H) | | | | 1 | CGU(R) | | 0.36 | |
| CUC(L) | 0.63 | | | CCC(P) | 0.87 | CAC(H) | | | | 1 | CGC(R) | | 0.99 | |
| CUA(L) | 2.48 | | | CCA(P) | 1.75 | CAA(Q) | | | | 1.88 | CGA(R) | | 1.93 | |
| CUG(L) | 0.3 | | | CCG(P) | 0.22 | CAG(Q) | | | | 0.12 | CGG(R) | | 0.72 | |
| AUU(I) | 1.08 | | | ACU(T) | 0.99 | AAU(N) | | | | 0.95 | AGU(S) | | 0.67 | |
| AUC(I) | 0.92 | | | ACC(T) | 1.3 | AAC(N) | | | | 1.05 | AGC(S) | | 1.41 | |
| AUA(M) | 1.61 | | | ACA(T) | 1.58 | AAA(K) | | | | 1.91 | AGA(*) | | 1 | |
| AUG(M) | 0.39 | | | ACG(T) | 0.13 | AAG(K) | | | | 0.09 | AGG(*) | | 1.48 | |
| GUU(V) | 1.21 | | | GCU(A) | 0.8 | GAU(D) | | | | 0.51 | GGU(G) | | 0.83 | |
| GUC(V) | 0.6 | | | GCC(A) | 1.66 | GAC(D) | | | | 1.49 | GGC(G) | | 1.19 | |
| GUA(V) | 1.94 | | | GCA(A) | 1.49 | GAA(E) | | | | 1.8 | GGA(G) | | 1.4 | |
| GUG(V) | 0.25 | | | GCG(A) | 0.05 | GAG(E) | | | | 0.2 | GGG(G) | | 0.57 | |
| ***L. punctate*** | | | | | | | | | | | | | | |
| Codon | RSCU | | | Codon | RSCU | Codon | | | | RSCU | Codon | | RSCU | |
| UUU(F) | 1.11 | | | UCU(S) | 1.22 | UAU(Y) | | | | 1.04 | UGU(C) | | 1.21 | |
| UUC(F) | 0.89 | | | UCC(S) | 1.07 | UAC(Y) | | | | 0.96 | UGC(C) | | 0.79 | |
| UUA(L) | 1.15 | | | UCA(S) | 1.74 | UAA(*) | | | | 1.37 | UGA(W) | | 1.01 | |
| UUG(L) | 0.82 | | | UCG(S) | 0.29 | UAG(*) | | | | 0.93 | UGG(W) | | 0.99 | |
| CUU(L) | 1.04 | | | CCU(P) | 1.43 | CAU(H) | | | | 1.12 | CGU(R) | | 0.93 | |
| CUC(L) | 0.88 | | | CCC(P) | 0.8 | CAC(H) | | | | 0.88 | CGC(R) | | 1.09 | |
| CUA(L) | 1.61 | | | CCA(P) | 1.6 | CAA(Q) | | | | 1.44 | CGA(R) | | 1.01 | |
| CUG(L) | 0.51 | | | CCG(P) | 0.17 | CAG(Q) | | | | 0.56 | CGG(R) | | 0.97 | |
| AUU(I) | 1.14 | | | ACU(T) | 1.35 | AAU(N) | | | | 1.08 | AGU(S) | | 0.52 | |
| AUC(I) | 0.86 | | | ACC(T) | 1.02 | AAC(N) | | | | 0.92 | AGC(S) | | 1.15 | |
| AUA(M) | 1.32 | | | ACA(T) | 1.28 | AAA(K) | | | | 1.56 | AGA(*) | | 0.87 | |
| AUG(M) | 0.68 | | | ACG(T) | 0.34 | AAG(K) | | | | 0.44 | AGG(*) | | 0.83 | |
| GUU(V) | 1.35 | | | GCU(A) | 0.78 | GAU(D) | | | | 1.06 | GGU(G) | | 0.85 | |
| GUC(V) | 0.52 | | | GCC(A) | 1.65 | GAC(D) | | | | 0.94 | GGC(G) | | 1.01 | |
| GUA(V) | 1.16 | | | GCA(A) | 1.39 | GAA(E) | | | | 1.42 | GGA(G) | | 1.23 | |
| GUG(V) | 0.97 | | | GCG(A) | 0.17 | GAG(E) | | | | 0.58 | GGG(G) | | 0.91 | |
| ***L. scutata*** | | | | | | | | | | | | | | |
| Codon | RSCU | | | Codon | RSCU | | Codon | RSCU | | | Codon | | RSCU | |
| UUU(F) | 1.06 | | | UCU(S) | 1.15 | | UAU(Y) | 1.02 | | | UGU(C) | | 0.96 | |
| UUC(F) | 0.94 | | | UCC(S) | 1.02 | | UAC(Y) | 0.98 | | | UGC(C) | | 1.04 | |
| UUA(L) | 1.24 | | | UCA(S) | 1.92 | | UAA(*) | 1.81 | | | UGA(W) | | 1.36 | |
| UUG(L) | 0.24 | | | UCG(S) | 0.25 | | UAG(*) | 0.69 | | | UGG(W) | | 0.64 | |
| CUU(L) | 0.98 | | | CCU(P) | 1.08 | | CAU(H) | 1.03 | | | CGU(R) | | 0.47 | |
| CUC(L) | 0.82 | | | CCC(P) | 0.78 | | CAC(H) | 0.97 | | | CGC(R) | | 0.75 | |
| CUA(L) | 2.36 | | | CCA(P) | 1.97 | | CAA(Q) | 1.78 | | | CGA(R) | | 1.98 | |
| CUG(L) | 0.36 | | | CCG(P) | 0.17 | | CAG(Q) | 0.22 | | | CGG(R) | | 0.8 | |
| AUU(I) | 0.97 | | | ACU(T) | 0.9 | | AAU(N) | 1.01 | | | AGU(S) | | 0.42 | |
| AUC(I) | 1.03 | | | ACC(T) | 1.33 | | AAC(N) | 0.99 | | | AGC(S) | | 1.23 | |
| AUA(M) | 1.62 | | | ACA(T) | 1.63 | | AAA(K) | 1.8 | | | AGA(*) | | 0.75 | |
| AUG(M) | 0.38 | | | ACG(T) | 0.14 | | AAG(K) | 0.2 | | | AGG(*) | | 0.75 | |
| GUU(V) | 0.84 | | | GCU(A) | 0.92 | | GAU(D) | 0.68 | | | GGU(G) | | 0.94 | |
| GUC(V) | 0.84 | | | GCC(A) | 1.44 | | GAC(D) | 1.32 | | | GGC(G) | | 1.05 | |
| GUA(V) | 1.89 | | | GCA(A) | 1.52 | | GAA(E) | 1.66 | | | GGA(G) | | 1.54 | |
| GUG(V) | 0.44 | | | GCG(A) | 0.13 | | GAG(E) | 0.34 | | | GGG(G) | | 0.47 | |
| ***P. steindachneri*** | | | | | | | | | | | | | | |
| Codon | RSCU | | | Codon | RSCU | Codon | | | | RSCU | Codon | | RSCU | |
| UUU(F) | 0.93 | | | UCU(S) | 1.16 | UAU(Y) | | | | 1.02 | UGU(C) | | 0.88 | |
| UUC(F) | 1.07 | | | UCC(S) | 1.28 | UAC(Y) | | | | 0.98 | UGC(C) | | 1.13 | |
| UUA(L) | 1.71 | | | UCA(S) | 2.43 | UAA(*) | | | | 2.22 | UGA(W) | | 1.42 | |
| UUG(L) | 0.57 | | | UCG(S) | 0.42 | UAG(*) | | | | 1.27 | UGG(W) | | 0.58 | |
| CUU(L) | 0.74 | | | CCU(P) | 1.08 | CAU(H) | | | | 0.99 | CGU(R) | | 0.47 | |
| CUC(L) | 0.59 | | | CCC(P) | 1.06 | CAC(H) | | | | 1.01 | CGC(R) | | 0.78 | |
| CUA(L) | 1.91 | | | CCA(P) | 1.67 | CAA(Q) | | | | 1.42 | CGA(R) | | 2.27 | |
| CUG(L) | 0.47 | | | CCG(P) | 0.19 | CAG(Q) | | | | 0.58 | CGG(R) | | 0.47 | |
| AUU(I) | 1.1 | | | ACU(T) | 1.01 | AAU(N) | | | | 0.87 | AGU(S) | | 0.16 | |
| AUC(I) | 0.9 | | | ACC(T) | 1.26 | AAC(N) | | | | 1.13 | AGC(S) | | 0.54 | |
| AUA(M) | 1.49 | | | ACA(T) | 1.49 | AAA(K) | | | | 1.69 | AGA(*) | | 0.26 | |
| AUG(M) | 0.51 | | | ACG(T) | 0.23 | AAG(K) | | | | 0.31 | AGG(*) | | 0.26 | |
| GUU(V) | 0.88 | | | GCU(A) | 1 | GAU(D) | | | | 0.93 | GGU(G) | | 0.77 | |
| GUC(V) | 0.75 | | | GCC(A) | 1.63 | GAC(D) | | | | 1.08 | GGC(G) | | 1.18 | |
| GUA(V) | 1.82 | | | GCA(A) | 1.23 | GAA(E) | | | | 1.47 | GGA(G) | | 1.18 | |
| GUG(V) | 0.55 | | | GCG(A) | 0.15 | GAG(E) | | | | 0.53 | GGG(G) | | 0.87 | |
| ***P. cantorii*** | | | | | | | | | | | | | | |
| Codon | RSCU | | | Codon | RSCU | Codon | | | RSCU | | | Codon | | RSCU |
| UUU(F) | 0.97 | | | UCU(S) | 1.08 | UAU(Y) | | | 0.89 | | | UGU(C) | | 0.97 |
| UUC(F) | 1.03 | | | UCC(S) | 1.35 | UAC(Y) | | | 1.11 | | | UGC(C) | | 1.03 |
| UUA(L) | 0.85 | | | UCA(S) | 1.54 | UAA(*) | | | 1.33 | | | UGA(W) | | 1.04 |
| UUG(L) | 0.63 | | | UCG(S) | 0.4 | UAG(*) | | | 0.82 | | | UGG(W) | | 0.96 |
| CUU(L) | 1.1 | | | CCU(P) | 1.26 | CAU(H) | | | 1.1 | | | CGU(R) | | 1.29 |
| CUC(L) | 0.91 | | | CCC(P) | 1.03 | CAC(H) | | | 0.9 | | | CGC(R) | | 0.86 |
| CUA(L) | 1.86 | | | CCA(P) | 1.53 | CAA(Q) | | | 1.41 | | | CGA(R) | | 0.96 |
| CUG(L) | 0.66 | | | CCG(P) | 0.18 | CAG(Q) | | | 0.59 | | | CGG(R) | | 0.89 |
| AUU(I) | 1.06 | | | ACU(T) | 1.42 | AAU(N) | | | 1 | | | AGU(S) | | 0.55 |
| AUC(I) | 0.94 | | | ACC(T) | 0.93 | AAC(N) | | | 1 | | | AGC(S) | | 1.08 |
| AUA(M) | 1.3 | | | ACA(T) | 1.3 | AAA(K) | | | 1.62 | | | AGA(*) | | 0.93 |
| AUG(M) | 0.7 | | | ACG(T) | 0.35 | AAG(K) | | | 0.38 | | | AGG(*) | | 0.93 |
| GUU(V) | 1.15 | | | GCU(A) | 0.93 | GAU(D) | | | 0.96 | | | GGU(G) | | 0.92 |
| GUC(V) | 0.68 | | | GCC(A) | 1.97 | GAC(D) | | | 1.04 | | | GGC(G) | | 1.3 |
| GUA(V) | 1.02 | | | GCA(A) | 1 | GAA(E) | | | 1.36 | | | GGA(G) | | 0.82 |
| GUG(V) | 1.15 | | | GCG(A) | 0.1 | GAG(E) | | | 0.64 | | | GGG(G) | | 0.96 |
| ***P. sinensis*** | | | | | | | | | | | | | | |
| Codon | RSCU | | Codon | | RSCU | Codon | | | | RSCU | Codon | | RSCU | |
| UUU(F) | 0.89 | | UCU(S) | | 0.76 | UAU(Y) | | | | 1.02 | UGU(C) | | 1.03 | |
| UUC(F) | 1.11 | | UCC(S) | | 1 | UAC(Y) | | | | 0.98 | UGC(C) | | 0.97 | |
| UUA(L) | 2.27 | | UCA(S) | | 2.92 | UAA(*) | | | | 2.77 | UGA(W) | | 1.86 | |
| UUG(L) | 0.2 | | UCG(S) | | 0.15 | UAG(*) | | | | 0.62 | UGG(W) | | 0.14 | |
| CUU(L) | 0.69 | | CCU(P) | | 0.63 | CAU(H) | | | | 0.8 | CGU(R) | | 0.41 | |
| CUC(L) | 0.54 | | CCC(P) | | 0.67 | CAC(H) | | | | 1.2 | CGC(R) | | 0.71 | |
| CUA(L) | 2.25 | | CCA(P) | | 2.63 | CAA(Q) | | | | 1.91 | CGA(R) | | 2.82 | |
| CUG(L) | 0.05 | | CCG(P) | | 0.06 | CAG(Q) | | | | 0.09 | CGG(R) | | 0.06 | |
| AUU(I) | 1.01 | | ACU(T) | | 0.63 | AAU(N) | | | | 0.78 | AGU(S) | | 0.32 | |
| AUC(I) | 0.99 | | ACC(T) | | 1.31 | AAC(N) | | | | 1.22 | AGC(S) | | 0.84 | |
| AUA(M) | 1.78 | | ACA(T) | | 2.01 | AAA(K) | | | | 1.93 | AGA(*) | | 0.31 | |
| AUG(M) | 0.22 | | ACG(T) | | 0.05 | AAG(K) | | | | 0.07 | AGG(*) | | 0.31 | |
| GUU(V) | 1.14 | | GCU(A) | | 0.85 | GAU(D) | | | | 0.81 | GGU(G) | | 0.95 | |
| GUC(V) | 0.44 | | GCC(A) | | 1.53 | GAC(D) | | | | 1.19 | GGC(G) | | 0.85 | |
| GUA(V) | 2.03 | | GCA(A) | | 1.55 | GAA(E) | | | | 1.83 | GGA(G) | | 1.73 | |
| GUG(V) | 0.39 | | GCG(A) | | 0.07 | GAG(E) | | | | 0.17 | GGG(G) | | 0.47 | |
| ***R. swinhoei*** | | | | | | | | | | | | | | |
| Codon | RSCU | | Codon | | RSCU | Codon | | | | RSCU | Codon | | RSCU | |
| UUU(F) | 1.2 | | UCU(S) | | 0.8 | UAU(Y) | | | | 1.01 | UGU(C) | | 1.04 | |
| UUC(F) | 0.8 | | UCC(S) | | 0.73 | UAC(Y) | | | | 0.99 | UGC(C) | | 0.96 | |
| UUA(L) | 0.99 | | UCA(S) | | 1.41 | UAA(*) | | | | 1.28 | UGA(W) | | 0.99 | |
| UUG(L) | 0.27 | | UCG(S) | | 0.21 | UAG(*) | | | | 0.44 | UGG(W) | | 1.01 | |
| CUU(L) | 1.41 | | CCU(P) | | 1.38 | CAU(H) | | | | 1.14 | CGU(R) | | 0.91 | |
| CUC(L) | 0.97 | | CCC(P) | | 1.08 | CAC(H) | | | | 0.86 | CGC(R) | | 1.13 | |
| CUA(L) | 1.9 | | CCA(P) | | 1.29 | CAA(Q) | | | | 1.78 | CGA(R) | | 0.91 | |
| CUG(L) | 0.46 | | CCG(P) | | 0.24 | CAG(Q) | | | | 0.22 | CGG(R) | | 1.06 | |
| AUU(I) | 1.14 | | ACU(T) | | 1.42 | AAU(N) | | | | 1.08 | AGU(S) | | 1.13 | |
| AUC(I) | 0.86 | | ACC(T) | | 1.03 | AAC(N) | | | | 0.92 | AGC(S) | | 1.72 | |
| AUA(M) | 1.3 | | ACA(T) | | 1.29 | AAA(K) | | | | 1.69 | AGA(*) | | 1.11 | |
| AUG(M) | 0.7 | | ACG(T) | | 0.27 | AAG(K) | | | | 0.31 | AGG(*) | | 1.17 | |
| GUU(V) | 1.68 | | GCU(A) | | 0.84 | GAU(D) | | | | 0.82 | GGU(G) | | 0.95 | |
| GUC(V) | 0.64 | | GCC(A) | | 1.98 | GAC(D) | | | | 1.18 | GGC(G) | | 1.24 | |
| GUA(V) | 1.33 | | GCA(A) | | 1.04 | GAA(E) | | | | 1.88 | GGA(G) | | 1.03 | |
| GUG(V) | 0.35 | | GCG(A) | | 0.15 | GAG(E) | | | | 0.12 | GGG(G) | | 0.78 | |
| ***T. triunguis*** | | | | | | | | | | | | | | |
| Codon | RSCU | | Codon | | RSCU | Codon | | | | RSCU | Codon | | RSCU | |
| UUU(F) | 0.69 | | UCU(S) | | 0.71 | UAU(Y) | | | | 0.87 | UGU(C) | | 1.13 | |
| UUC(F) | 1.31 | | UCC(S) | | 1.26 | UAC(Y) | | | | 1.13 | UGC(C) | | 0.88 | |
| UUA(L) | 0.77 | | UCA(S) | | 1.97 | UAA(*) | | | | 1.68 | UGA(W) | | 1.23 | |
| UUG(L) | 0.49 | | UCG(S) | | 0.31 | UAG(*) | | | | 0.43 | UGG(W) | | 0.77 | |
| CUU(L) | 0.87 | | CCU(P) | | 1.02 | CAU(H) | | | | 0.85 | CGU(R) | | 0.83 | |
| CUC(L) | 0.83 | | CCC(P) | | 1.04 | CAC(H) | | | | 1.15 | CGC(R) | | 0.71 | |
| CUA(L) | 2.54 | | CCA(P) | | 1.69 | CAA(Q) | | | | 1.69 | CGA(R) | | 1.67 | |
| CUG(L) | 0.5 | | CCG(P) | | 0.25 | CAG(Q) | | | | 0.31 | CGG(R) | | 0.79 | |
| AUU(I) | 0.78 | | ACU(T) | | 1.04 | AAU(N) | | | | 0.84 | AGU(S) | | 0.57 | |
| AUC(I) | 1.22 | | ACC(T) | | 1.39 | AAC(N) | | | | 1.16 | AGC(S) | | 1.18 | |
| AUA(M) | 1.64 | | ACA(T) | | 1.36 | AAA(K) | | | | 1.72 | AGA(*) | | 1 | |
| AUG(M) | 0.36 | | ACG(T) | | 0.21 | AAG(K) | | | | 0.28 | AGG(*) | | 0.9 | |
| GUU(V) | 1.12 | | GCU(A) | | 0.69 | GAU(D) | | | | 0.81 | GGU(G) | | 0.67 | |
| GUC(V) | 0.45 | | GCC(A) | | 1.9 | GAC(D) | | | | 1.19 | GGC(G) | | 1.2 | |
| GUA(V) | 1.53 | | GCA(A) | | 1.26 | GAA(E) | | | | 1.52 | GGA(G) | | 1.2 | |
| GUG(V) | 0.9 | | GCG(A) | | 0.15 | GAG(E) | | | | 0.48 | GGG(G) | | 0.93 | |

**Table S6. Comparison of anticodons of tRNAs within 13 Trionychidae species.** *nn= N. nigricans, nf= N. formosa, af= A. ferox, as= A. spinifera, ci= C. indica, ds= D. subplana, lp= L. punctata, lc= L. scutata, pst= P. steindachneri, pc= P. cantorii, psi= P. sinensis, rs= R. swinhoei, tt= T. triunguis.*

| **Locus** | ***Nn*** | ***nf*** | ***ds*** | ***pst*** | ***psi*** | ***rs*** | ***as*** | ***af*** | ***tt*** | ***Ci*** | ***Pc*** | ***Ls*** | ***lp*** |
| --- | --- | --- | --- | --- | --- | --- | --- | --- | --- | --- | --- | --- | --- |
| *trnF* | GAA | GAA | GAA | GAA | GAA | GAA | GAA | GAA | GAA | GAA | GAA | GAA | GAA |
| *trnV* | TAC | TAC | TAC | TAC | TAC | TAC | TAC | TAC | TAC | TAC | TAC | TAC | TAC |
| *trnL2* | TAA | TAA | TAA | TAA | TAA | TAA | TAA | TAA | TAA | TAA | TAA | TAA | TAA |
| *trnI* | GAT | GAT | GAT | GAT | GAT | GAT | GAT | GAT | GAT | GAT | GAT | GAT | GAT |
| *trnQ* | TTG | TTG | TTG | TTG | TTG | TTG | TTG | TTG | TTG | TTG | TTG | TTG | TTG |
| *trnM* | CAT | CAT | CAT | CAT | CAT | CAT | CAT | CAT | CAT | CAT | CAT | CAT | CAT |
| *trnW* | TCA | TCA | TCA | TCA | TCA | TCA | TCA | TCA | TCA | TCA | TCA | TCA | TCA |
| *trnA* | TGC | TGC | TGC | TGC | TGC | TGC | TGC | TGC | TGC | TGC | TGC | TGC | TGC |
| *trnN* | GTT | GTT | GTT | GTT | GTT | GTT | GTT | GTT | GTT | GTT | GTT | GTT | GTT |
| *trnC* | GCA | GCA | GCA | GCA | GCA | GCA | GCA | GCA | GCA | GCA | GCA | GCA | GCA |
| *trnY* | GTA | GTA | GTA | GTA | GTA | GTA | GTA | GTA | GTA | GTA | GTA | GTA | GTA |
| *trnS2* | TGA | TGA | TGA | TGA | TGA | TGA | TGA | TGA | TGA | TGA | TGA | TGA | TGA |
| *trnD* | GTC | GTC | GTC | GTC | GTC | GTC | GTC | GTC | GTC | GTC | GTC | GTC | GTC |
| *trnK* | TTT | TTT | TTT | TTT | TTT | TTT | TTT | TTT | TTT | TTT | TTT | TTT | TTT |
| *trnG* | TCC | TCC | TCC | TCC | TCC | TCC | TCC | TCC | TCC | TCC | TCC | TCC | TCC |
| *trnR* | TCG | TCG | TCG | TCG | TCG | TCG | TCG | TCG | TCG | TCG | TCG | TCG | TCG |
| *trnH* | GTG | GTG | GTG | GTG | GTG | GTG | GTG | GTG | GTG | GTG | GTG | GTG | GTG |
| *trnS1* | GCT | GCT | GCT | GCT | GCT | GCT | GCT | GCT | GCT | GCT | GCT | GCT | GCT |
| *trnL1* | TAG | TAG | TAG | TAG | TAG | TAG | TAG | TAG | TAG | TAG | TAG | TAG | TAG |
| *trnE* | TTC | TTC | TTC | TTC | TTC | TTC | TTC | TTC | TTC | TTC | TTC | TTC | TTC |
| *trnT* | TGT | TGT | TGT | TGT | TGT | TGT | TGT | TGT | TGT | TGT | TGT | TGT | TGT |
| *trnP* | TGG | TGG | TGG | TGG | TGG | TGG | TGG | TGG | TGG | TGG | TGG | TGG | TGG |

**Table S7. The Ka/Ks values of 13 PCGs in 13 complete mitogenomes of Trionychidae species.** *nn= N. nigricans, nf= N. formosa, af= A. ferox, ci= C. indica, ds= D. subplana, lp= L. punctata, pst= P. steindachneri, pc= P. cantorii, psi= P. sinensis, rs= R. swinhoei, tt= T. triunguis.*

| PCGs | nn/af | nn/ci | nn/ds | nn/lp | nn/nf | nn/pst | nn/pc | nn/psi | nn/rs | nn/tt | Average | STDEV |
| --- | --- | --- | --- | --- | --- | --- | --- | --- | --- | --- | --- | --- |
| *atp6* | 0.12035 | 0.144703 | 0.148501 | 0.15943 | 0.053453 | 0.121202 | 0.10567 | 0.145044 | 0.118417 | 0.132877 | 0.124965 | 0.028569 |
| *atp8* | 0.39249 | 0.391975 | 0.352138 | 0.211807 | 0.233874 | 0.286904 | 0.325141 | 0.220954 | 0.352138 | 0.2294 | 0.299682 | 0.068291 |
| *cox1* | 0.407309 | 0.363775 | 0.35376 | 0.409635 | 0.44133 | 0.470774 | 0.406086 | 0.311149 | 0.402854 | 0.385133 | 0.39518 | 0.042711 |
| *cox2* | 0.0313 | 0.063193 | 0.052086 | 0.059117 | 0.015399 | 0.030997 | 0.050875 | 0.03138 | 0.049366 | 0.041667 | 0.042538 | 0.014248 |
| *cox3* | 0.052019 | 0.033349 | 0.06336 | 0.057713 | 0.033043 | 0.020862 | 0.050685 | 0.028007 | 0.032687 | 0.049635 | 0.042136 | 0.013496 |
| *cytb* | 0.972594 | 1.06762 | 1.416094 | 1.056253 | 0.825893 | 0.881514 | 1.203597 | 1.123336 | 1.056366 | 0.908901 | 1.051217 | 0.163671 |
| *nad1* | 1.042665 | 0.994813 | 1.143533 | 1.272877 | 1.525157 | 0.690207 | 1.143853 | 1.04834 | 1.007194 | 1.337811 | 1.120645 | 0.21455 |
| *nad2* | 0.787984 | 0.578965 | 0.588118 | 0.598762 | 0.768191 | 0.518491 | 0.708026 | 0.577083 | 0.761598 | 0.753738 | 0.664096 | 0.095769 |
| *nad3* | 0.388835 | 0.270646 | 0.32389 | 0.250483 | 0.192691 | 0.266098 | 0.248919 | 0.366499 | 0.327001 | 0.207072 | 0.284214 | 0.061923 |
| *nad4* | 0.87727 | 0.829384 | 0.816057 | 0.920383 | 0.840734 | 0.742605 | 0.777155 | 0.655451 | 0.737714 | 0.763719 | 0.796047 | 0.072796 |
| *nad4l* | 1.095267 | 1.424324 | 0.598891 | 1.282431 | 1 | 1.205479 | 1.051813 | 0.759525 | 1.431122 | 1.449833 | 1.129869 | 0.27424 |
| *nad5* | 0.394023 | 0.530873 | 0.406988 | 0.406988 | 0.405749 | 0.382139 | 0.410959 | 0.410162 | 0.43949 | 0.406955 | 0.419433 | 0.039583 |
| *nad6* | 0.25584 | 0.260926 | 0.133065 | 0.230594 | 0.081355 | 0.182567 | 0.226373 | 0.144934 | 0.185091 | 0.278831 | 0.197958 | 0.060622 |
